# Control of Paternally Expressed Imprinted *UPWARD CURLY LEAF1*, a Gene Encoding an F-Box Protein That Regulates CURLY LEAF Polycomb Protein, in the *Arabidopsis* Endosperm

**DOI:** 10.1371/journal.pone.0117431

**Published:** 2015-02-17

**Authors:** Cheol Woong Jeong, Guen Tae Park, Hyein Yun, Tzung-Fu Hsieh, Yang Do Choi, Yeonhee Choi, Jong Seob Lee

**Affiliations:** 1 School of Biological Sciences, Seoul National University, Seoul, Korea; 2 Plants for Human Health Institute & Department of Plant and Microbial Biology, North Carolina State University, Kannapolis, North Carolina, United State of America; 3 Department of Agricultural Biotechnology, Seoul National University, Seoul, Korea; Wuhan University, CHINA

## Abstract

Genomic imprinting, an epigenetic process in mammals and flowering plants, refers to the differential expression of alleles of the same genes in a parent-of-origin-specific manner. In *Arabidopsis*, imprinting occurs primarily in the endosperm, which nourishes the developing embryo. Recent high-throughput sequencing analyses revealed that more than 200 loci are imprinted in *Arabidopsis*; however, only a few of these imprinted genes and their imprinting mechanisms have been examined in detail. Whereas most imprinted loci characterized to date are maternally expressed imprinted genes (MEGs), *PHERES1* (*PHE1*) and *ADMETOS* (*ADM*) are paternally expressed imprinted genes (PEGs). Here, we report that *UPWARD CURLY LEAF1* (*UCL1*), a gene encoding an E3 ligase that degrades the CURLY LEAF (CLF) polycomb protein, is a PEG. After fertilization, paternally inherited *UCL1* is expressed in the endosperm, but not in the embryo. The expression pattern of a *β-glucuronidase* (*GUS*) reporter gene driven by the *UCL1* promoter suggests that the imprinting control region (ICR) of *UCL1* is adjacent to a transposable element in the *UCL1* 5′-upstream region. Polycomb Repressive Complex 2 (PRC2) silences the maternal *UCL1* allele in the central cell prior to fertilization and in the endosperm after fertilization. The *UCL1* imprinting pattern was not affected in paternal PRC2 mutants. We found unexpectedly that the maternal *UCL1* allele is reactivated in the endosperm of *Arabidopsis* lines with mutations in cytosine DNA *METHYLTRANSFERASE 1* (*MET1*) or the DNA glycosylase *DEMETER* (*DME*), which antagonistically regulate CpG methylation of DNA. By contrast, maternal *UCL1* silencing was not altered in mutants with defects in non-CpG methylation. Thus, silencing of the maternal *UCL1* allele is regulated by both MET1 and DME as well as by PRC2, suggesting that divergent mechanisms for the regulation of PEGs evolved in *Arabidopsis*.

## Introduction

*Arabidopsis* seeds contain three tissues that have distinct parental genome contributions, namely 1) the diploid embryo, which is the diploid fertilization product of the maternal and paternal genomes, 2) the triploid endosperm, which is the fertilization product of the diploid maternal and haploid paternal genomes, and 3) the seed coat, which is of diploid maternal origin [1,2]. Although communication and interaction between these tissues are critical for proper seed development, the underlying mechanisms are largely unknown [3–5]. The unequal parental genetic contribution affects seed development due to genome dosage and parent-of-origin effects.

The parent-of-origin-dependent differential allelic expression of a single gene is known as genomic imprinting. Thus, imprinted genes are predominantly expressed from either the maternal or paternal allele. Genomic imprinting occurs primarily in mammals and flowering plants. In *Arabidopsis*, imprinting takes place mainly in the endosperm, the tissue that nourishes the developing embryo [6]. Several theories have been proposed to explain the evolution of imprinting, the most popular of which is that imprinting arose due to parental conflict over resource allocation to the embryo [7–9]. Another theory for the evolution of imprinting is that it is required to limit the gene dosage of key genes during early development [10,11]. The genomic imbalance between maternal and paternal dosages affects seed and embryo development in both plants and mammals. An increase in paternal dosage leads to an increase in placental or endosperm growth, whereas an increase in maternal dosage has the opposite effect [12,13].

*MEDEA (MEA)*, the first imprinted gene to be reported in *Arabidopsis*, was described more than a decade ago [14]. Recently, thanks to next generation sequencing of expression libraries or RNAs at the whole genome level, more than 200 loci were found to be imprinted in *Arabidopsis* [15–18]. However, the mechanisms by which differential allelic expression is regulated have been studied for only a few imprinted genes. The expression and silencing of *MEA*, *FERTILIZATION INDEPENDENT SEED2* (*FIS2*), *FLOWERING WAGENINGEN* (*FWA*), *PHERES1* (*PHE1*), and *ADMETOS* (*ADM*) have been characterized [14,19–21]. While *MEA*, *FIS2*, and *FWA* are maternally expressed imprinted genes (MEGs), *PHE1* and ADM are paternally expressed imprinted genes (PEGs). The maternal alleles of *MEA*, *FIS2*, and *FWA* are activated in the central cell of the female gametophyte. Their activation requires sequential steps involving two antagonistic genes; DNA *METHYLTRANSFERASE 1* (*MET1*), which adds a methyl group to a cytosine base, and *DEMETER* (*DME*), which functions as a demethylase. During megagametogenesis, the transcription of *MET1* is down-regulated by RETINOBLASTOMA-RELATED 1 (RBR1) and its binding partner MULTICOPY SUPPRESSOR OF IRA1 (MSI1), resulting in partial passive hypomethylation [22]. Then, *DME* is expressed in the central cell of the mature female gametophyte [23], where it removes residual methyl cytosine from its target genes [24,25]. Thus, the maternal alleles of *MEA*, *FIS2*, and *FWA* are expressed in the central cell before fertilization. After fertilization, the maternal alleles are epigenetically maintained in the hypomethylated state and are continuously expressed in the endosperm. Methylated cytosines can be directly removed by DME and REPRESSOR OF SILENCING 1 (ROS1) and the resulting abasic sites are replaced with unmethylated cytosines through the base excision repair (BER) pathway [24,26]. Thus, activation of the maternal alleles of *MEA*, *FIS2*, and *FWA* is controlled by DNA methylation. Accordingly, loss-of-function mutations in *DME* result in at least partial hypermethylation and silencing of the maternal alleles of *MEA*, *FIS2*, and *FWA*. On the other hand, silenced paternal *FIS2* and *FWA* alleles are derepressed when *met1* mutants are inherited paternally, indicating that silencing of the paternal *FIS2* and *FWA* alleles is controlled by MET1 [20,21]. By contrast, the paternal *MEA* allele is silenced not by DNA methylation, but by the MEA-containing FIS-Polycomb Repressive Complex 2 (FIS-PRC2), and is thus self-regulated [24,27,28]. In the case of the PEG *PHE1*, repression of the maternal allele is controlled by FIS-PRC2 and requires the unmethylated 3′-repeat region of the *PHE1* locus [19,29]. This repeat region located distantly downstream of *PHE1* is hypermethylated in the expressed paternal allele. Another PEG, *ADM*, which belongs to the diverse family of molecular chaperones called J-domain proteins and determines seed viability in paternal excess interploidy hybridizations, was identified as a target gene of the FIS-PRC2 in the endosperm [30].

Although imprinting evolved independently in mammals and in flowering plants [31], the imprinting mechanisms in the *Arabidopsis* endosperm and in the mammalian placenta or embryo are partially parallel. In mammals, several imprinted genes are particularly important for placental development [32] and most of the imprinted genes are located in clusters in the imprinting control regions (ICRs), which are enriched in CpG islands and subjected to methylation [33]. Whereas imprinted genes are organized in large chromosomal clusters in mammals, imprinted plant genes appear to occur as singletons [31]. Recent efforts to identify the DNA sequences responsible for imprinted expression in *Arabidopsis* revealed that plant ICRs are located close to the imprinted loci [19,29].

Previously, we reported that *UPWARD CURLY LEAF1* (*UCL1*), which encodes an E3 ligase that regulates CURLY LEAF (CLF) protein in *Arabidopsis*, is expressed exclusively in the endosperm [34]. Here, we investigated whether the expression of *UCL1* is regulated by imprinting. To answer this question, we examined the allele-specific expression of endogenous *UCL1* as well as that of *UCL1* reporter transgenes. We found that the ICR of *UCL1* was adjacent to the transposable element in the 5′-upsteam region of *UCL1*. FIS-PRC2 is required for the repression of the maternal allele of *UCL1*. In addition, repression of the maternal *UCL1* allele is associated with DNA methylation near the ICR. Mutations in both *MET1* and *DME* caused derepression of the silenced maternal *UCL1* allele. These results provide new insight into the epigenetic mechanisms that maintain imprinting of *UCL1* in the *Arabidopsis* endosperm.

## Results

### *UCL1* is a paternally expressed imprinted gene in the *Arabidopsis* endosperm

**Fig 1:**
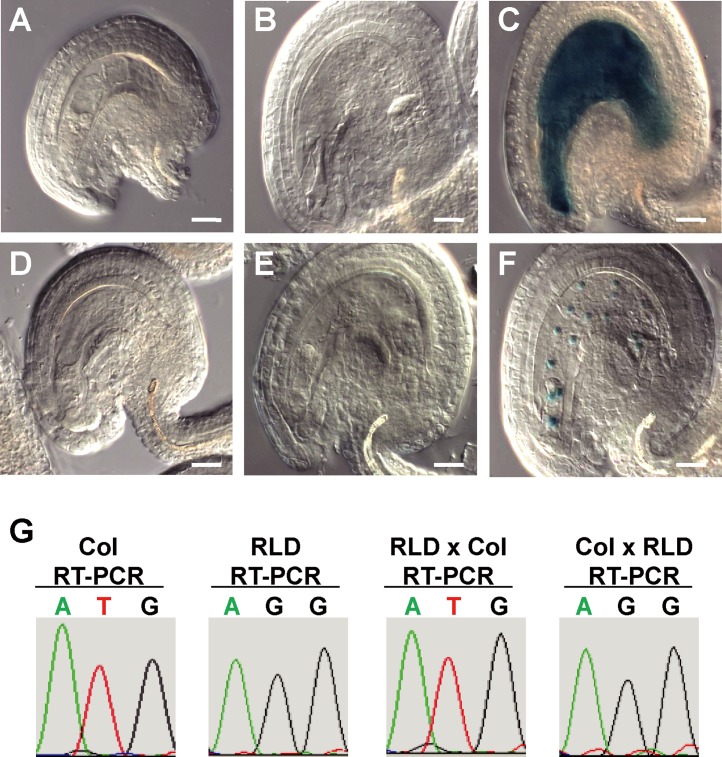
*UCL1* is paternally expressed in the endosperm. (A-C) Ovule and seeds derived from reciprocal crosses between the *UCL1_4*.*1k*::*GUS* transgenic plant and Col-0 wild type. (A) Expression of the maternally derived *UCL1_4*.*1k*::*GUS* transgene in a wild-type ovule after emasculation. B) Expression of the maternally derived *UCL1_4*.*1k*::*GUS* transgene in a wild-type seed at 1 day after pollination (DAP). (C) Expression of the paternally derived *UCL1_4*.*1k*::*GUS* transgene in the wild-type seed at 1 DAP. (D-F) Ovules and seeds resulting from reciprocal crosses between a *UCL1_4*.*1k*::*UCL1*:*GUS* transgenic plant and the wild type. (D) Expression of the maternally derived *UCL1_4*.*1k*::*UCL1*:*GUS* transgene in a wild-type ovule after emasculation. (E) Expression of the maternally derived *UCL1_4*.*1k*::*UCL1*:*GUS* transgene in a wild-type seed at 1 DAP. (F) Expression of the paternally derived *UCL1_4*.*1k*::*GUS* transgene in a wild-type seed at 1 DAP. Scale bars: 20 μm. (G) Sequencing chromatograms of RT-PCR products of *UCL1* showing allele-specific expression at a polymorphic site indicated in S1 Fig. Endosperm RNA was prepared in samples derived from reciprocal crosses between Col-0 and RLD ecotypes.

We previously reported that *UCL1* is substantially expressed in flowers, young stamens, and developing seeds [34]. Cytoplasmic GUS activity driven by the *UCL1* promoter was detected in young stamens of floral stages 9 to 10 and then decreased significantly before fertilization. Strong GUS activity was observed in the endosperm after fertilization [34]. To investigate whether *UCL1* expression shows parent-of-origin specificity, we performed reciprocal crosses between *UCL1_4*.*1k*::*GUS* (transcriptional fusion) transgenic plants and Col-0 wild type plants (Fig. 1A–1C). GUS activity was not detected in the female gametophyte of the transgenic plants before fertilization (Fig. 1A) or in the developing seeds of *UCL1_4*.*1k*::*GUS* transgenic plants pollinated by the Col-0 wild-type plants (Fig. 1B). In the seeds of wild-type plants pollinated by the transgenic plants, however, cytoplasmic *GUS* expression was detected in the endosperm (Fig. 1C).

Next, we reciprocally crossed *UCL1_4*.*1k*::*UCL1*:*GUS* (translational fusion) transgenic plants with Col-0 wild-type plants (Fig. 1D–1F). GUS activity was detected neither in the female gametophyte (Fig. 1D) nor in the developing endosperm of the transgenic plants pollinated by wild-type plants (Fig. 1E). In contrast, when wild-type plants were pollinated by *UCL1_4*.*1k*::*UCL1*:*GUS* transgenic plants, GUS activity was detected in proliferating endosperm nuclei, but not in the embryo (Fig. 1F). Thus, these observations indicate that only the paternal allele of the *UCL1* transgene is expressed in the developing endosperm.

To test whether endogenous *UCL1* expression also depends on its parent-of-origin, single nucleotide polymorphisms (SNPs) among different *Arabidopsis* ecotypes were used to differentiate parent-specific *UCL1* transcripts. While Col-0, L*er*, and En-2 ecotypes have identical sequences within the *UCL1* coding region, RLD and C24 ecotypes have polymorphic sites (S1 Fig). RT-PCR analysis was performed using RNAs extracted from the developing seeds resulting from reciprocal crosses between Col-0 and RLD. The sequencing chromatogram of the amplification products showed a single peak of the SNP corresponding to the paternal allele (Fig. 1G). Taken together, these results indicate that *UCL1* is expressed only from the paternally inherited allele and thus that *UCL1* is a paternally expressed imprinted gene (PEG) in the endosperm.

### The 5′-upstream region controls *UCL1* imprinting

**Fig 2:**
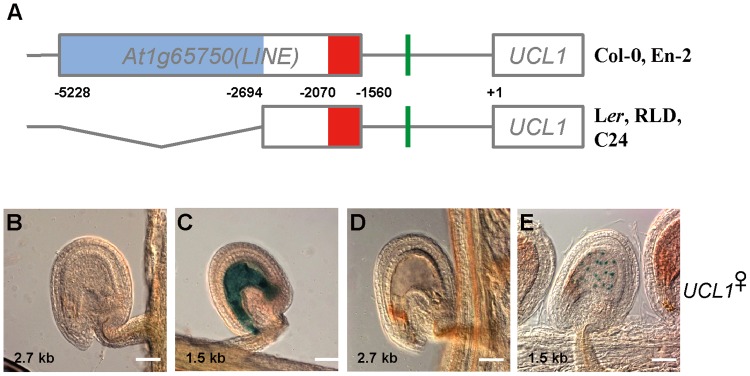
Structure of the *UCL1* locus in different *Arabidopsis* ecotypes. (A) Overview of the *UCL1* locus in different *Arabidopsis* ecotypes. The blue and red boxes indicate distinct TEs in the *ATLINE1_1* family of the LINE/L1 superfamily in *At1065750*. L*er*, RLD, and C24 do not include the long *ATLINE1_1* TE, whereas Col-0 and En-2 do. The numbers are in base pairs (bp) from the translation start site of *UCL1*. (B) Expression of the maternally derived *UCL1_2*.*7k*::*GUS* transgene in a wild-type seed at 1 DAP. (C) Expression of the maternally derived *UCL1_1*.*5k*::*GUS* transgene in a wild-type seed at 1 DAP. (D) Expression of the maternally derived *UCL1_2*.*7k*::*UCL1*:*GUS* transgene in a wild-type seed at 1 DAP. (E) Expression of the maternally derived *UCL1_1*.*5k*::*UCL1*:*GUS* transgene in a wild-type seed at 1 DAP. Scale bars: 50 μm.

According to the TAIR annotation, *At1g65750*, which is located upstream of the *UCL1* (*At1g65740*) locus, encodes a non-LTR retrotransposon (LINE). Using the RepeatMasker program (http://www.repeatmasker.org/cgi-bin/WEBRepeatMasker), we identified two *ATLINE1_1* transposable elements (TEs) in the Col-0 ecotype, namely 1) a long one (blue box) between 2.7 and 5.2 kb upstream and 2) a short one (red box) 1.5 and 2.0 kb upstream of the *UCL1* translation start site (Fig. 2A). Interestingly, while the Col-0 and En-2 ecotypes contained the two *ATLINE1_1* TEs, L*er*, RLD, and C24 possess only the short *ATLINE1_1* TE, which is closer to the *UCL1* coding region (Fig. 2A). In addition, simple repeat sequences (Fig. 2A, green bar) were predicted at the 1.0-kb upstream region using the RepeatMasker program.

Because the transcriptional and translational fusions of the *GUS* transgenes driven by the *UCL1* promoter recapitulated the imprinted expression of the endogenous *UCL1* gene, we analyzed *GUS* expression using different lengths of the *UCL1* promoter to identify the region necessary for *UCL1* imprinting. To examine the activity of a *UCL1* promoter fragment lacking the two *ATLINE1_1* TEs, the 1.5-kb fragment upstream of *UCL1* was transcriptionally fused to *GUS* (*UCL1_1*.*5k*::*GUS)* (S2D and S2L Fig) and the transgenic plants were reciprocally crossed with Col-0 wild-type plants. Cytoplasmic GUS activity was detected in the central cell of the female gametophyte before fertilization in the *UCL1_1*.*5k*::*GUS* transgenic plants (S2D Fig), but not in the female gametophyte of the *UCL1_4*.*1k*::*GUS* plants (Fig. 1A and S2B Fig). After fertilization, the cytoplasmic GUS signal was detected not only in the self-fertilized seeds of *UCL1_1*.*5k*::*GUS* transgenic plants (S2L Fig) but also in the seeds of wild-type plants pollinated by *UCL1_1*.*5k*::*GUS* transgenic plants (S3L Fig) or of *UCL1_1*.*5k*::*GUS* transgenic plants pollinated by wild-type plants (Fig. 2C and S3D Fig).

**Fig 3:**
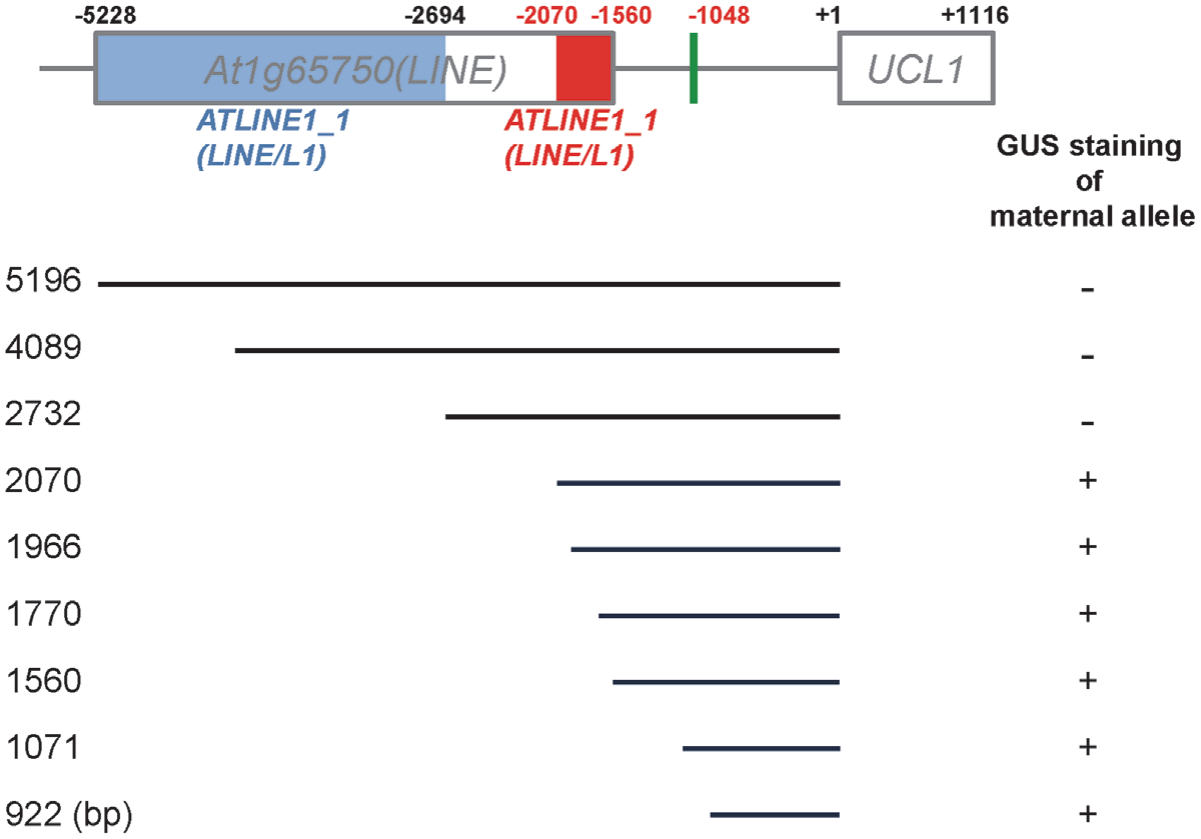
The ICR of *UCL1* is located between two LINE/L1 TEs. In the diagram of the promoter region of *UCL1*, the blue and red boxes indicate the distinct TEs in the *ATLINE1_1* family of the LINE/L1 superfamily in *At1065750*. The numbers to the left of the lines indicate the size of the promoters (in bp) fused to the *GUS* transgene. Transgenic plants carrying the *GUS* transgene fused to various lengths of the *UCL1* promoter were generated and the expression of the maternally derived transgenes was analyzed in seeds at 1 DAP, similarly as in Fig. 2.

We also generated *UCL1_1*.*5k*::*UCL1*:*GUS* (translational fusion) transgenic plants and reciprocally crossed these plants with Col-0 wild type plants. Consistent with the data from the *UCL1_1*.*5k*::*GUS* plants, GUS activity was detected in the central cell nucleus prior to fertilization (S2H Fig). After fertilization, both the maternally and paternally derived transgene showed GUS activity in the proliferating endosperm nuclei (Fig. 2E and S3H and S3P Fig). The bi-allelic expression of the *GUS* transgenes containing the 1.5-kb *UCL1* promoter fragment suggests that the imprinting control region (ICR) of *UCL1* is not present in the region 1.5 kb upstream of *UCL1*.

By contrast, no maternal *GUS* expression was detected in the female gametophyte before fertilization or in the endosperm of *UCL1_2*.*7k*::*GUS* plants pollinated with the Col-0 wild-type plants (Figs. 2B and S2C and S3C). GUS activity was detected in the endosperm when the *UCL1_2*.*7k*::*GUS* transgene was inherited paternally (S3K Fig), suggesting that the *UCL1_2*.*7k*::*GUS* transgene is imprinted and that the region 2.7 kb upstream of *UCL1* contains the ICR of *UCL1*.

We also generated transgenic plants carrying the *UCL1_2*.*7k*::*UCL1*:*GUS* translational fusion. Consistent with the transcriptional *UCL1_2*.*7k*::*GUS* data, nuclear GUS activity was detected in the endosperm only when the transgene was inherited paternally (Fig. 2D and S3G and S3O Fig). Taken together, these results suggest that the ICR of *UCL1* is located in the region between 2.7 kb and 1.5 kb upstream of the *UCL1* translation start site and that this region is necessary for the repression of the *UCL1* maternal allele in the central cell before fertilization and in the endosperm after fertilization.

TE sequences are thought to be highly methylated due to the silencing of the invading foreign DNA [35–37]. One short *ATLINE1_1* TE is located in the 2.0-kb upstream region of the *UCL1* locus (Fig. 2A, red box); thus, it is possible that this short *ATLINE1_1* TE might be the target of methylation and function as the ICR of *UCL1*. To test this possibility, we generated constructs containing various lengths of the *UCL1* promoter, corresponding to 2.0 kb, 1.9 kb, 1.7 kb, and 1.0 kb from the translation start site, fused to *GUS* and examined GUS staining in developing seeds of the corresponding transgenic plants after crossing with wild-type pollen. Surprisingly, all transgenic plants showed bi-allelic expression of the *GUS* transgenes (Figs. 3 and S4). These results clearly demonstrate that the ICR that underlies the maternal repression of *UCL1* is located in the 5′-upstream region of this gene, between 2.7 kb and 2.0 kb from the translation start site, but that the *cis*-element(s) responsible for default bi-allelic expression of *UCL1* is contained in the 1.0-kb upstream sequence.

### PRC2 controls the silencing of maternal *UCL1*

FIS-PRC2, containing the four core polycomb group proteins MEA, FIS2, FIE, and MSI1, regulates not only seed development, but also genomic imprinting in *Arabidopsis* [19,20,24,28]. Because *UCL1* is a paternally expressed and maternally silenced imprinted gene in the endosperm after fertilization, we tested whether FIS-PRC2 is involved in maternal *UCL1* repression.

**Fig 4:**
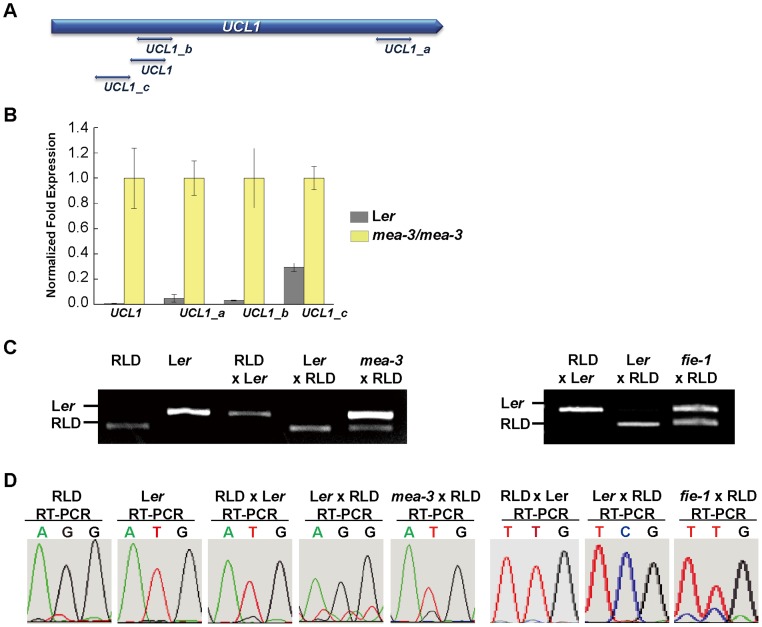
MEA proteins are required for the repression of the maternal allele of *UCL1*. (A) Location of qRT-PCR primer sets used to detect the expression of *UCL1*. (B) Comparison of the expression levels of *UCL1* in wild-type L*er* and *mea-3* endosperm at 3 DAP. The expression of *UCL1* in the *mea-3*/*mea-3* mutant was set to 1 and the error bar represents the standard deviation of three independent samples. (C) Analysis of the allele-specific expression of *UCL1* using a CAPS marker. RT-PCR analysis was performed on RNA isolated from the endosperms of RLD females crossed with L*er* males, L*er* females crossed with RLD males, and *mea-3* or *fie-1* females (L*er* background) crossed with RLD males. These products were digested with *Eco*RI. The L*er* allele shows a 276 bp band, whereas the RLD allele was cut into a 222-bp band after *Eco*RI digestion. (D) Sequencing chromatograms of the RT-PCR products of *UCL1* at the distinguished SNP regions showing allele-specific expression. RNAs were isolated from endosperms resulting from reciprocal crosses between RLD and L*er* ecotypes and in crosses between the female *mea-3* or *fie-1* mutant and the male RLD.

Firstly, we analyzed *UCL1* expression in *mea-3* homozygous mutant seeds. Real-time quantitative reverse transcription-polymerase chain reaction (qRT-RCR) revealed a strong increase in expression in four regions of *UCL1* in the *mea-3* homozygous mutant seeds (Fig. 4A and Fig. 4B). This suggests that the silenced maternal *UCL1* allele might be de-repressed in *mea-3* seeds.

Secondly, using RT-PCR and cleaved amplified polymorphic sequence (CAPS) markers of different ecotypes, we analyzed the expression of the maternally and paternally derived *UCL1* alleles. Consistent with the transgene data, maternal *UCL1* was silenced and paternal *UCL1* was expressed in the developing seeds. However, when we used *mea-3* homozygous plants or *fie-1* heterozygous plants pollinated by RLD wild-type plants, the maternal *UCL1* allele was activated, resulting in bi-allelic expression in the developing seeds (Fig. 4C). This finding shows that MEA and FIE are indeed required for the repression of the *UCL1* maternal allele. To confirm the allele-specific expression of endogenous *UCL1*, we verified the sequence chromatogram of distinguished SNPs between L*er* and RLD. The sequencing chromatogram showed that pollination of the maternally inherited *mea* or *fie* mutant with RLD pollen caused activation of the maternal *UCL1* allele, which was silenced in L*er* wild-type plants (Fig. 4D). Interestingly, not only the Col-0 and En-2 ecotypes, which carry the 5.2-kb upstream sequences containing the two TEs, but also the L*er*, RLD, and C24 ecotypes, which have only the 2.7-kb upstream region containing the short TE, show imprinting (Fig. 1G, Fig. 2A, Fig. 4C and 4D).

**Fig 5:**
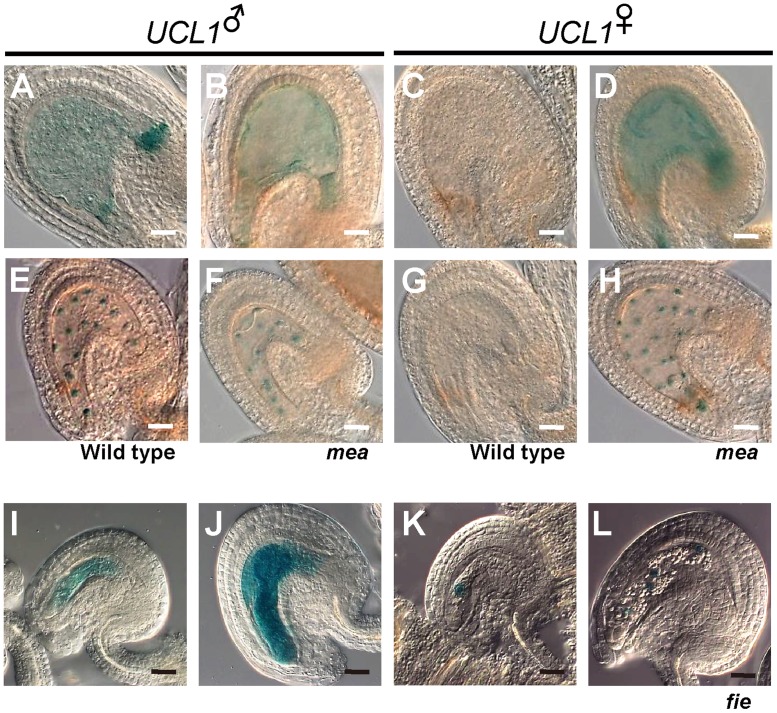
MEA polycomb proteins are required for the repression of the maternal allele of *UCL1*. (A) Expression of the paternally derived *UCL1_4*.*1k*::*GUS* transgene in a wild-type seed at 1 DAP. (B) Expression of the paternally derived *UCL1_4*.*1k*::*GUS* transgene in a *mea-3* mutant seed at 1 DAP. (C) Expression of the maternally derived *UCL1_4*.*1k*::*GUS* transgene in a wild-type seed at 1 DAP. (D) Expression of the maternally derived *UCL1_4*.*1k*::*GUS* transgene in a *mea-3* mutant seed at 1 DAP. (E) Expression of the paternally derived *UCL1_4*.*1k*::*UCL1*:*GUS* transgene in a wild-type seed at 1 DAP. (F) Expression of the paternally derived *UCL1_4*.*1k*::*UCL1*:*GUS* transgene in a *mea-3* mutant seed at 1 DAP. (G) Expression of the maternally derived *UCL1_4*.*1k*::*UCL1*:*GUS* transgene in a wild-type seed at 1 DAP. (H) Expression of the maternally derived *UCL1_4*.*1k*::*UCL1*:*GUS* transgene in a *mea-3* mutant seed at 1 DAP. Scale bars: 20 μm. (I-L) The ovule and autonomously developing endosperm of plants that were hemizygous for the *GUS* transgene and heterozygous for *fie-1* after emasculation. (I) Expression of the *UCL1_4*.*1k*::*GUS* transgene in a *fie-1* mutant ovule at 1 day after emasculation. (J) Expression of the *UCL1_4*.*1k*::*GUS* transgene in an autonomously developing endosperm in the *fie-1* mutant at 2 days after emasculation. (K) Expression of the *UCL1_4*.*1k*::*UCL1*:*GUS* transgene in a *fie-1* mutant ovule at 1 day after emasculation. (L) Expression of the *UCL1_4*.*1k*::*UCL1*:*GUS* transgene in an autonomously developing endosperm in the *fie-1* mutant at 2 days after emasculation. Scale bars: 20 μm.

Thirdly, we examined the expression of the maternal *UCL1_4*.*1k*::*GUS* and *UCL1_4*.*1k*::*UCL1*:*GUS* transgenes in the wild-type and *mea-3* background. To determine whether the paternal *UCL1* expression pattern is affected by mutation of *MEA*, we pollinated wild-type stigmas with pollen derived from plants hemizygous for the *GUS* transgenes and heterozygous for the *mea-3* mutation. No differences were observed in the seeds, indicating that the absence of *MEA* in the paternally derived genome does not affect *UCL1* imprinting in the endosperm (Fig. 5B compared to 5A and 5F compared to 5E). Furthermore, inheritance of the *fie-1* mutant through the male parent did not alter the endogenous *UCL1* imprinting pattern (S5 Fig). Conversely, when wild-type pollen was used to pollinate plants hemizygous for the *GUS* transgenes and heterozygous for *mea-3*, the maternally inherited *UCL1_4*.*1k*::*GUS* and *UCL1_4*.*1k*::*UCL1*:*GUS* transgenes were de-repressed, suggesting that MEA is required for silencing of the maternally derived *UCL1* allele in the endosperm (Fig. 5D compared to 5C and 5H compared to 5G).

The initiation of endosperm development before fertilization is repressed by the FIS-PRC2 complex [38]. Among the polycomb group proteins constituting this complex, the *fie* mutant showed a stronger phenotype of diploid central cell proliferation, resulting in a higher percentage of autonomous endosperm development in the silique when fertilization was blocked [39,40]. To elucidate whether maternal *UCL1* expression in the central cell of the female gametophyte is repressed by FIE, plants that were heterozygous for the *fie-1* mutation and hemizygous for the *UCL1_4*.*1k*::*GUS* or *UCL1_4*.*1k*::*UCL1*:*GUS* transgene were emasculated and *GUS* expression was examined in the ovules. Whereas no *GUS* expression was detected in the female gametophyte either one- or two-days after emasculation when the transgenes were in the wild-type background, some female gametophytes of the *fie-1* heterozygous mutants showed GUS signals after emasculation. Cytoplasmic GUS was detected in the central cell and in the autonomous endosperm after emasculation in the plants heterozygous for the *fie-1* mutation and hemizygous for the *UCL1_4*.*1k*::*GUS* transgene (Fig. 5I and 5J). Likewise, the GUS signal was detected not only in the nucleus of the diploid central cell, but also in the nuclei of the dividing central cells of the autonomous endosperm of plants heterozygous for *fie-1* and hemizygous for the *UCL1_4*.*1k*::*UCL1*:*GUS* transgene when emasculated (Fig. 5K to 5L).

These results demonstrate that functional MEA and FIE which are components of a FIS-PRC2 complex is responsible for the repression of maternal *UCL1* expression in the central cell of the female gametophyte prior to fertilization and in the endosperm after fertilization.

### DNA methylation affects *UCL1* imprinting

**Fig 6:**
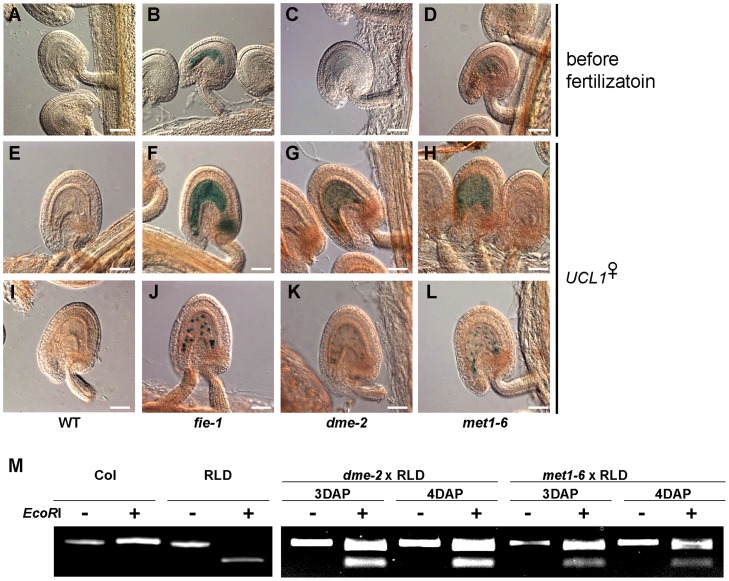
DNA methylation is also relevant to maternal *UCL1* silencing. (A-D) Ovules after emasculation. (A) Expression of the maternally derived *UCL1_4*.*1k*::*GUS* transgene in a wild-type ovule. (B) Expression of the maternally derived *UCL1_4*.*1k*::*GUS* transgene in a *fie-1* mutant ovule. (C) Expression of the maternally derived *UCL1_4*.*1k*::*GUS* transgene in a *dme-2* mutant female gametophyte. (D) Expression of the maternally derived *UCL1_4*.*1k*::*GUS* transgene in a *met1–6* mutant ovule. (E-L) Seeds from plants hemizygous for the *GUS* transgene and heterozygous for *fie-1*, *dme-2*, or *met1–6*. The *fie-1*, *dme-2*, and *met1–6* mutants were used as females in crosses with wild-type pollen to characterize the expression of the *UCL1* maternal allele. (E) Expression of the maternally derived *UCL1_4*.*1k*::*GUS* transgene in a wild-type seed at 1 DAP. (F) Expression of the maternally derived *UCL1_4*.*1k*::*GUS* transgene in a *fie-1* mutant seed at 1 DAP. (G) Expression of the maternally derived *UCL1_4*.*1k*::*GUS* transgene in a *dme-2* mutant seed at 1 DAP. (H) Expression of the maternally derived *UCL1_4*.*1k*::*GUS* transgene in a *met1–6* mutant seed at 1 DAP. (I) Expression of the maternally derived *UCL1_4*.*1k*::*UCL1*:*GUS* transgene in a wild-type seed at 1 DAP. (J) Expression of the maternally derived *UCL1_4*.*1k*::*UCL1*:*GUS* transgene in a *fie-1* mutant seed at 1 DAP. (K) Expression of the maternally derived *UCL1_4*.*1k*::*UCL1*:*GUS* transgene in a *dme-2* mutant seed at 1 DAP. (L) Expression of the maternally derived *UCL1_4*.*1k*::*UCL1*:*GUS* transgene in a *met1–6* mutant seed at 1 DAP. Scale bars: 50 μm. (M) Analysis of the allele-specific expression of *UCL1* using a CAPS marker. Endosperm RNAs were prepared from the female *met1–6* or *dme-2* mutant (Col background) crossed with the male RLD plant at both 3 DAP and 4 DAP. The RT-PCR products were analyzed before and after *Eco*RI digestion.

DNA methylation and histone methylation are important epigenetic mechanisms regulating genomic imprinting in animals and plants [20,23,41]. While the maternally expressed imprinted *FWA* and *FIS2* genes have a differentially methylated region (DMR) in their promoter regions, the paternally expressed imprinted *PHE1* gene has a DMR in the 3′-downstream region. To test whether *UCL1* imprinting is regulated by DNA methylation, we emasculated plants that were heterozygous for the *dme-2* mutation and hemizygous for the *UCL1_4*.*1k*::*GUS* or *UCL1_4*.*1k*::*UCL1*:*GUS* transgene. Plants hemizygous for the *UCL1_4*.*1k*::*GUS* or *UCL1_4*.*1k*::*UCL1*:*GUS* transgene in the wild-type background were used as a negative control (Fig. 6A, 6E, and 6I) and plants heterozygous for the *fie-1* mutation and hemizygous for the *UCL1_4*.*1k*::*GUS* or *UCL1_4*.*1k*::*UCL1*:*GUS* transgene were used as a positive control (Fig. 6B, 6F, and 6J). After emasculation, maternal GUS activity was detected in the central cell of the *dme-2* female gametophyte (Fig. 6C). After pollination with wild-type pollen, maternal GUS activity was detected in the endosperm of the *dme-2* mutant (Fig. 6G and 6K). The maternal *UCL1* allele was silenced by FIS2-PRC2 (Figs. 4 and 5). Given that DME is required for the activation of the *MEA* and *FIS2* maternal alleles [20,23,24], the maternal *UCL1* expression in the *dme-2* mutant might be due to the lack of FIS-PRC2 in the central cell and the endosperm rather than to the hypermethylation of the endosperm DNA.

We also investigated *UCL1* imprinting in the *met1–6* mutant [42], which exhibits global hypomethylation of the genome [43]. We emasculated plants heterozygous for the *met1–6* mutation and hemizygous for the *UCL1_4*.*1k*::*GUS* or *UCL1_4*.*1k*::*UCL1*:*GUS* transgene, and examined the resulting GUS activity in ovules. Maternal GUS expression was detected in the central cell of the *met1–6* mutant female gametophyte (Fig. 6D). We also performed crosses of plants hemizygous for the *GUS* transgene and heterozygous for *met1–6* as females with wild-type pollen. The maternally inherited *UCL1_4*.*1k*::*GUS* and *UCL1_4*.*1k*::*UCL1*:*GUS* transgenes in the maternal *met1–6* mutants were de-repressed in the endosperm (Fig. 6H and 6L). By contrast, we could not detect any GUS signal in crosses using plants that were heterozygous for *argonaute 4* (*ago4–1*), *rna-dependent rna polymerase 2* (*rdr2–1*), or *dicer-like 3* (*dcl3–1*), which are involved in asymmetric methylation through RNA-dependent DNA methylation (RdDM) (S6 Fig). Taken together, these results imply that the repression of the *UCL1* maternal allele is not related to the asymmetric RdDM pathway, but is related to symmetric CpG DNA methylation. A possible mechanism whereby DNA methylation mediates *UCL1* imprinting will be discussed below.

**Fig 7:**
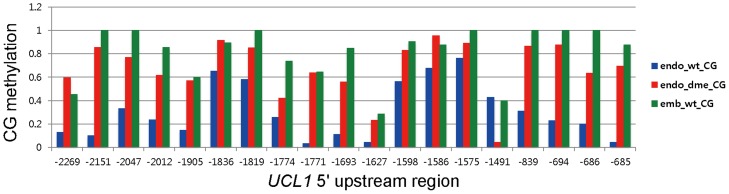
CpG methylation patterns of the 5′ upstream region of *UCL1* in the endosperm and embryo. Only CpG sites with fractional CpG methylation that is significantly different between the embryo and endosperm, and between the wild-type endosperm and *dme-2* endosperm are shown. Numbers on the x-axis represent CpG site positions (in bp) relative to the *UCL1* translational start site.

To further our understanding of *UCL1* methylation patterns, we analyzed publicly available CpG methylation data in wild-type and *dme-2* endosperms [36]. CpG methylation is significantly lower in the wild-type endosperm than in the wild-type embryo or *dme-2* endosperm, indicating that the *UCL1* promoter is demethylated by DME in the central cell (Fig. 7 and S1 Table; p<0.05 in both wild-type endosperm—*dme* endosperm, and wild-type endosperm—wild-type embryo comparisons). Hypomethylation of the *UCL1* promoter is more significant around the short *ATLINE1_1* TE, and extends into the ICR region, which is located between 2.0 kb and 2.7 kb upstream of the translation start site of *UCL1*. The CpG methylation profile of the *UCL1* promoter is consistent with the observation that DME activity is required for silencing of the *UCL1* maternal allele, presumably by allowing FIS-PRC2 to bind to and establish silencing in the ICR. By contrast, no significant differences in non-CpG methylation were found between the embryo and endosperm, or between the wild-type and *dme* endosperm in the *UCL1* promoter, supporting the observation that mutations in the RdDM pathway do not affect *UCL1* imprinting.

## Discussion

### The imprinting control region of *UCL1* is located in the 5′ upstream region

*GUS* transgene expression and endogenous *UCL1* expression as analyzed using allele-specific SNPs indicate that *UCL1* is an imprinted gene in the endosperm after fertilization (Fig. 1). Whereas the paternal *UCL1* allele is expressed in the endosperm, the maternal allele is silenced. To identify the imprinting control region (ICR) of *UCL1*, we first analyzed the flanking sequences near the *UCL1* coding region. Although TAIR annotation suggests that *At1g65750* has a single non-LTR retrotransposon (LINE), which is located upstream of *UCL1* (*At1g65740*), *At1g65750* is likely composed of two *ATLINE1_1* transposable elements (TEs), according to the RepeatMasker program. Interestingly, the number of *ATLINE1_1* TEs is not identical in different ecotypes. While both *ATLINE1_1* TEs are present in the Col-0 and En-2 ecotypes, only the short *ATLINE1_1* TE, which is closer to the *UCL1* coding region, is in the L*er*, RLD, and C24 ecotypes (Fig. 2). However, *UCL1* still showed imprinting in the L*er* and RLD ecotypes (Fig. 4), suggesting that the absence of the long *ATLINE1_1* TE in the further upstream region does not affect *UCL1* imprinting and that the ICR is located within the region 2.7 kb upstream of the translation start site of *UCL1*. Deletion analysis of the *UCL1* promoter confirmed that the ICR of *UCL1* is located between 2.0 kb and 2.7 kb upstream of *UCL1* (Fig. 3).

Imprinted genes are often associated with natural parasitic elements such as TEs and tandem repeats [29,44]. Imprinting has been proposed to arise as a byproduct of silencing of invading foreign DNA [36,45]. TEs sometimes have negative effects on genome integrity; however, they also provide a source for genetic and epigenetic diversity during evolution. TEs have been implicated as targets of methylation by various mechanisms [46–48]. DNA methylation silences these potentially damaging DNA elements and also the neighboring genes. Imprinted genes in *Arabidopsis* are frequently in close proximity to differentially methylated regions (DMRs), most of which correspond to short TEs [6,16,35,36]. Regulation of gene expression by DNA methylation could be selected for if imprinting of these genes mediates the parental conflict or gene dosage balance in the triploid endosperm [49]. Because the short *ATLINE1_1* TE is present in the *UCL1* upstream region and CpG residues of the *ATLINE1–1* TE are indeed methylated in the embryo (Fig. 7) [36], the short *ATLINE1–1* TE is a likely *UCL1* ICR candidate. However, the *GUS* transgene driven by the region 2.0 kb upstream of *UCL1* showed bi-allelic expression in the presence of the short *ATLINE1–1* TE. Therefore, the ICR of *UCL1* is considered to be located between 2.0 kb and 2.7 kb upstream of the translation start site of *UCL1*. Consistently, the repeat sequences in the 1.0-kb upstream region did not affect *UCL1* imprinting, indicating that the repeat sequences are not the ICR of *UCL1* (Fig. 3).

ICRs and DMRs have been identified in plant imprinted genes [31]. The DMR of *PHE1* is located in the 3′ downstream region that contains triple repeats [29]. The *MEA* ICR was reported to be located in the 5′ upstream region [50]. The SINE-related sequence located in the 5′ upstream region of *FWA* was identified as being sufficient for imprinting [44,51]. Although the *MEA* ICR was reported to be independent of DME and MET1, other ICR-like sequences are controlled by DNA methylation. Nonetheless, the effects of DNA methylation on ICR-like sequences are different for each imprinted gene. Lack of sequence homology in the plant ICR-like sequences suggests that divergent imprinting mechanisms exist in plants, and that these mechanisms require sequence-specific imprinting factors.

### PRC2 and DNA methylation control *UCL1* imprinting

MEA, FIS2, FIE, and MSI1 form the seed-specific FIS-PRC2 and are required for proper seed development. *MEA* and *FIS2* are maternally expressed imprinted genes in the endosperm [14,20]. Whereas *FIS2* imprinting is solely regulated by DNA methylation, *MEA* imprinting is more complex; maternal *MEA* is activated by DME DNA glycosyalse and paternal *MEA* is silenced by maternally-expressed MEA-containing PRC2 [20,24]. Thus, maternal *MEA* activation is established by DME-mediated DNA demethylation in the central cell and, in turn, the maternally expressed MEA-containing PRC2 silences its own paternal *MEA* allele. In contrast, *UCL1* is a paternally expressed imprinted gene in the endosperm. The expression of the maternal *UCL1* allele was de-repressed in the *mea* and *fie* mutants (Figs. 4 and 5), demonstrating that maternal *UCL1* is silenced by FIS-PRC2.

DNA methylation is involved in silencing of many imprinted genes, including *FWA* [52]. Maternal *FWA* is demethylated and activated by DME, and paternal *FWA* is hypermethylated and repressed in the endosperm [21]. A short interspersed nuclear element (SINE)-related sequence in the *FWA* upstream region is sufficient for *FWA* imprinting and shows a differential methylation pattern depending on the parental origin [21,44,51]. When the wild-type stigma is pollinated with hypomethylated mutant pollen, such as *met1* or *ddm1*, the silenced paternal *FWA* allele is derepressed in the endosperm. By contrast, the repressed *PHE1* maternal allele is hypomethylated and the expressed *PHE1* paternal allele is hypermethylated at its 3′ repeat region in the endosperm [29]. DNA methylation is necessary for the paternal expression of *PHE1*. Therefore, DNA methylation of the imprinted genes can produce opposite outcomes, i.e., activation or repression. It has been suggested that FIS-PRC2 preferentially binds to hypomethylated regions of DNA and then silences nearby genes [29,37]. While FIS-PRC2 binds to and represses the hypomethylated maternal *PHE1* allele, the active paternal *PHE1* allele is hypermethylated in the differentially methylated 3′ repeat region, and this blocks FIS-PRC2 binding.

Genome-wide DNA methylation profiles in the endosperm revealed that TEs and repetitive sequences are hypomethylated in the endosperm as compared to the embryo [35]. Methylome data revealed that the *UCL1* promoter region containing the short *ATLINE1_1* TE has much lower levels of CpG methylation in the endosperm than in the embryo, and that hypomethylation in the endosperm is DME-dependent, indicating that the *UCL1* promoter is demethylated by DME in the central cell prior to fertilization (Fig. 7). The *UCL1* maternal allele is de-repressed in the *dme* mutant female gametophyte and endosperm, suggesting that proper demethylation is required for establishing maternal *UCL1* silencing. Silencing and maintenance of repressed maternal *UCL1* also depend on FIS-PRC2, which is consistent with the notion that demethylation of tandem repeats downstream of maternal *PHE1* allows binding of FIS-PRC2 and subsequent silencing [15,29,37].

Although DNA demethylation is a global phenomenon, only selected sequences are targeted by FIS-PRC2, suggesting that DNA demethylation is necessary, but not sufficient for targeting of FIS-PRC2 [53]. The ICR of *UCL1* is located further upstream of the short *ATLINE1_1* TE, whereas CpG hypomethylation occurs at the AT-rich region and at the short *ATLINE1_1* TE (Fig. 7). Therefore, the short *ATLINE1_1* TE is probably demethylated by DME, but the sequence 700 bp upstream of *ATLINE1_1* TE may be required for targeting of FIS-PRC2, functioning as a polycomb response element, and spreading H3K27me3 for stable repression. This hypothesis is supported by the observation that the 2.0-kb promoter fragment containing the short TE drives biallelic expression of the transgene (Fig. 3). Future work is needed to further define the ICR and identify its associated epigenetic marks, such as H3K27me3.

Unexpectedly, the maternal *UCL1* allele was also activated when a *met1* mutant was crossed with wild-type pollen. This can possibly be explained by titration of PRC2 binding; *met1* mutants cause global hypomethylation in the genome and if a certain or fixed amount of FIS-PRC2 is available, FIS-PRC2 can move to the newly exposed FIS-PRC2-binding sites in *met1* mutants. Thus, the previously silenced *UCL1* maternal allele is activated. This hypothesis can be tested by comparing the results of chromosome immunoprecipitation (ChIP) assays using PRC2 antibody or H3K27me3 antibody in *met1* or the wild type. Another possibility is that a cryptic promoter causes de-repression in the *met1* mutant. Interestingly, the maternal *PHE1* allele is not reactivated in the endosperm when the *met1* mutant was crossed with wild-type pollen. Thus, *PHE1* and *UCL1* imprinting is individually fine-tuned, although both genes are PEGs that are regulated by DMRs and FIS-PRC2 binding.

Why the paternal *UCL1* allele is not expressed in the mature male gametophyte [34] remains unclear. One possible explanation is that the endosperm-specific activator (or transcription factor) that is absent in pollen induces paternal *UCL1* expression in the endosperm. Alternatively, the unknown repressor in the mature pollen grain that is absent in the endosperm may inhibit the expression of the paternal *UCL1* allele in the male gametophyte.

### Control of paternal *UCL1* imprinting is distinct from that of *PHE1*

Although many PEGs have been identified by genome-wide approaches [15,16], *PHE1* is the only one that has been thoroughly examined in *Arabidopsis* [29,54]. *PHE1* imprinting depends on a distantly located region downstream of the *PHE1* locus. *PHE1* expression depends on the presence of methylation of this downstream region, whereas *PHE1* repression is associated with the absence of methylation at this region [54]. *PHE1* imprinting is controlled by direct tandem repeats in the downstream region [54]. On the other hand, FIS-PRC2 binding to the *PHE1* promoter region and DNA demethylation of the 3′ region of *PHE1* are both necessary and sufficient for stable maternal *PHE1* repression [54]. However, although maternal *UCL1* repression requires DME-mediated demethylation and binding of FIS-PRC2, expression of the paternal *UCL1* allele does not seem to be regulated by DNA methylation, as is *PHE1*. Furthermore, the ICR located in the 700-bp region of the *UCL1* promoter appears not to be involved in paternal *UCL1* expression, because the fragment 1 kb upstream of the translation start site of *UCL1* confers biallelic expression of *UCL1* in the endosperm (Fig. 3 and S4 Fig). While the maternal *PHE1* allele was not reactivated in mutants that are defective in DNA methylation [29], the maternal *UCL1* allele was de-repressed in the *met1–6* mutant (Fig. 6H, 6L, and 6M). Therefore, our study provides insights into the divergent imprinting mechanisms that arose during the evolution of flowering plants.

## Materials and Methods

### Plant materials and growth conditions

All plants used in this study were *Arabidopsis thaliana* in the Columbia (Col-0) ecotype, except for the *mea-3* [55] and *fie-1* [40] mutants, which were isolated in the L*er* ecotype. The *met1–6* mutant [42] used in this study was in the Col-*gl* ecotype and only the first *met1–6* homozygous mutants generated from a self-pollinated *met1–6* heterozygote that had never been homozygous were used. Plants were grown in Sunshine Mix 5 under long-day (16 h/8 h) conditions at 23°C. Col-0 ecotype plants were used for *Agrobacterium*-mediated transformation by the floral dip method [56].

### Recombinant plasmid construction and *Agrobacterium* transformation

To construct the *UCL1_4*.*1k*::*GUS* or *UCL1_4*.*1k*::*UCL1*:*GUS* transgene, PCR-amplified fragments containing the *UCL1* upstream region (–4089 to –1, relative to the translational start site) were generated with primer sets JCW616/JCW619 and JCW616/JCW620 (S2 Table) using wild type Col-0 genomic DNA as template, and subcloned into the *Sal*I and *BamH*I sites of the *pBI101* vector. To construct the *UCL1_5*.*2k*::*GUS*, *UCL1_2*.*7k*::*GUS*, *UCL1_1*.*5k*::*GUS* or *UCL1_5*.*2k*::*UCL1*:*GUS*, *UCL1_2*.*7k*::*UCL1*:*GUS*, and *UCL1_1*.*5k*::*UCL1*:*GUS* transgenes described in S3 and S4 Figs, PCR-amplified fragments containing the *UCL1* upstream region (–5196 to –1, –2732 to –1, and–1560 to –1, relative to the translational start site) were generated with primer sets JCW615, JCW617, and JCW618 using wild type Col-0 genomic DNA, and subcloned into the *Sal*I and *Bam*HI sites of the *pBI101* vector. To construct *UCL1_2*.*0k*::*GUS*, *UCL1_1*.*9k*::*GUS*, *UCL1_1*.*7k*::*GUS*, *UCL1_1*.*0k*::*GUS*, and *UCL1_0*.*9k*::*GUS*, PCR-amplified fragments containing the *UCL1* upstream region (–2070 to –1, –1966 to –1, –1770 to –1, –1071 to –1, and -922 to —1, relative to the translational start site) were generated with primer sets shown in S2 Table. For translational *GUS* fusions with the lengths of *UCL1* promoters shown in S3 Fig, the primer JCW620, which lacks a stop codon, was used as the reverse primer with different forward primers (S2 Table). The seeds of transgenic plants were screened on half-strength MS medium containing 50 μg/ml kanamycin sulfate, and the resistant T1 seedlings were transferred to soil.

### Histochemical GUS assay

The histochemical GUS assay was performed as previously described [34]. Briefly, the samples were fixed in 90% acetone and then soaked in staining solution containing 1 mg/ml X-GlcA (5-bromo-4-chloro-3-indolyl glucuronide) in 50 mM Na_2_HPO_4_ (pH 7.2), 5 mM potassium ferricyanide/ferrocyanide, and 0.1% Triton X-100 at 37°C overnight after washing in the same staining solution without X-GlcA. The next day, the staining buffer was removed, and the samples were mounted in clearing solution (2.5 g chloral hydrate, 0.7 ml H_2_O, 0.3 ml glycerol) or 1×PBS for microscopy.

### Microscopy

The mounted tissue samples were observed on a Zeiss Axio Imager A1 microscope with a DIC filter and photographed using an AxioCamHRc camera (Carl Zeiss).

### RT-PCR and quantitative real time qRT-PCR

Total RNA was extracted from tissue ground in liquid nitrogen using the RNeasy Mini Kit (Qiagen) and the RNase-free DNase Kit (Qiagen), according to the manufacturers’ instructions, and messenger RNA was extracted from ground tissue with liquid nitrogen using the Dynabeads mRNA DIRECT^TM^ Kit (Invitrogen DYNAL), according to the manufacturer’s instructions. Following DNase-I treatment, 2 μg of total RNA or the entire amount of mRNA isolated from each sample was reverse transcribed into cDNA using oligo(dT) primer (18 mer) and the RevertAid^TM^ First Strand cDNA Synthesis Kit (Fermentas). Real time qRT-PCR was performed as previously described [34]. One tenth of the final volume of the reverse transcription (RT) product was used for each PCR reaction. Expression of gene transcripts was quantitated by iQ5 (Bio-Rad) and data were analyzed using the iCycler iQ system software (Bio-Rad). All PCR mixtures contained 10 μl of iQ SYBR Green Supermix (Bio-Rad), 0.5 μl of forward and reverse primers (10 μM), and 5 μl of 50 times diluted RT product per well. Samples were normalized against *β2 tubulin* or *actin* levels.

### Sequencing of *At1g65750* and *At1g65760* in different *Arabidopsis* ecotypes

To amplify *At1g65750* and *At1g65760* in different *Arabidopsis* ecotypes, the primer sets JCW637/638 and JCW639/640 (S2 Table) were used. Agarose gel electrophoresis revealed that a band of 4.6 kb was amplified from the genomic DNA of Col-0 and En-2. By contrast, the corresponding band amplified from L*er*, RLD, and C24 was 2.0 kb. Each band was excised from the gel and purified using the NucleoSpin Gel Clean-up Kit (MACHEREY-NAGEL) for sequencing.

### Allele-specific expression analysis

To analyze allele-specific *UCL1* expression, SNPs were identified (S1 Fig). To detect SNPs among the Col-0, En-2, L*er*, RLD, and C24 ecotypes, DNA fragments amplified using the JCW118/JCW481 primer set (S2 Table) were sequenced and the sequences were aligned using ClustalW. To detect allele-specific expression, the products amplified with primer sets JCW641/JCW642 from cDNA prepared from the seeds after crossing were sequenced with the same primers. The amplified DNA was digested with *Eco*RI to detect expressed alleles. Within the polymorphic site, *Eco*RI-digestion of the RT-PCR products amplified from RLD and C24 produced 222-bp and 54-bp fragments, in contrast to the 276-bp uncut products from L*er*, En-2, and Col-0. The *Eco*RI-digested amplified products were analyzed on 4% agarose gels.

### Analysis of the CpG methylation pattern of the 5′-upstream region of *UCL1*

Publicly available methylation data sets for wild-type embryo (Col-0×L*er*) and endosperm (Col-0×L*er*), and *dme-2* mutant endosperm (*dme-2* Col-0×L*er*) were used to analyze *UCL1* promoter methylation patterns [36]. Methylation patterns in all three sequence contexts (i.e., CpG, CHG, and CHH) around the *UCL1* locus were retrieved and pair-wise comparisons between embryo and endosperm, and between wild-type and *dme-2* endosperm were made. Only cytosines with significant differences in both comparisons (Fisher’s exact test p<0.05) were selected.

## Supporting Information

S1 Fig Identification of SNPs in the *UCL1* coding region in *Arabidopsis* ecotypes. Identical nucleotides in different ecotypes are indicated with asterisks and distinguishable SNPs are indicated with dots and red boxes. The Col-0, L*er*, and En-2 ecotypes have identical *UCL* sequences, while the RLD and C24 ecotypes have polymorphic sites.
(PDF)

S2 Fig Expression of the *GUS* transgene driven by *UCL1* promoter regions of various lengths. (A-H) Ovules expressing *GUS* transgenes driven by various lengths of the *UCL1* promoter after emasculation. (A) Expression of the maternally derived *UCL1_5*.*2k*::*GUS* transgene in a wild-type ovule. (B) Expression of the maternally derived *UCL1_4*.*1k*::*GUS* transgene in a wild-type ovule. (C) Expression of the maternally derived *UCL1_2*.*7k*::*GUS* transgene in a wild-type ovule. (D) Expression of the maternally derived *UCL1_1*.*5k*::*GUS* transgene in a wild-type ovule. (E) Expression of the maternally derived *UCL1_5*.*2k*::*UCL1*:*GUS* transgene in a wild-type ovule. (F) Expression of the maternally derived *UCL1_4*.*1k*::*UCL1*:*GUS* transgene in a wild-type ovule. (G) Expression of the maternally derived *UCL1_2*.*7k*::*UCL1*:*GUS* transgene in a wild-type ovule. (H) Expression of the maternally derived *UCL1_1*.*5k*::*UCL1*:*GUS* transgene in a wild-type ovule. (I-P) Seeds expressing *GUS* transgenes driven by various lengths of the *UCL1* promoter after self- pollination. (I) Expression of the *UCL1_5*.*2k*::*GUS* transgene in a wild-type seed at 1 DAP. (J) Expression of the *UCL1_4*.*1k*::*GUS* transgene in a wild-type seed at 1 DAP. (K) Expression of the *UCL1_2*.*7k*::*GUS* transgene in a wild-type seed at 1 DAP. (L) Expression of the *UCL1_1*.*5k*::*GUS* transgene in a wild-type seed at 1 DAP. (M) Expression of the *UCL1_5*.*2k*::*UCL1*:*GUS* transgene in a wild-type seed at 1 DAP. (N) Expression of the *UCL1_4*.*1k*::*UCL1*:*GUS* transgene in a wild-type seed at 1 DAP. (O) Expression of the *UCL1_2*.*7k*::*UCL1*:*GUS* transgene in a wild-type seed at 1 DAP. (P) Expression of the *UCL1_1*.*5k*::*UCL1*:*GUS* transgene in a wild-type seed at 1 DAP. Scale bars: 50 μm.
(PDF)

S3 Fig Allele-specific expression of the *GUS* transgene driven by *UCL1* promoter regions of various lengths. (A-D, I-L) **S**eeds of reciprocal crosses between the *UCL1*::*GUS* transgenic plant and Col-0 wild type. (E-H, M-P) Seeds of reciprocal crosses between the *UCL1_4*.*1k*::*UCL1*:*GUS* transgenic plant and the Col-0 wild type. (A) Expression of the maternally derived *UCL1_5*.*2k*::*GUS* transgene in a wild-type seed at 1 DAP. (B) Expression of the maternally derived *UCL1_4*.*1k*::*GUS* transgene in a wild-type seed at 1 DAP. (C) Expression of the maternally derived *UCL1_2*.*7k*::*GUS* transgene in a wild-type seed at 1 DAP. (D) Expression of the maternally derived *UCL1_1*.*5k*::*GUS* transgene in a wild-type seed at 1 DAP. (E) Expression of the maternally derived *UCL1_5*.*2k*::*UCL1*:*GUS* transgene in a wild-type seed at 1 DAP. (F) Expression of the maternally derived *UCL1_4*.*1k*::*UCL1*:*GUS* transgene in wild-type seed at 1 DAP. (G) Expression of the maternally derived *UCL1_2*.*7k*::*UCL1*:*GUS* transgene in a wild-type seed at 1 DAP. (H) Expression of the maternally derived *UCL1_1*.*5k*::*UCL1*:*GUS* transgene in a wild-type seed at 1 DAP. (I) Expression of the paternally derived *UCL1_5*.*2k*::*GUS* transgene in a wild-type seed at 1 DAP. (J) Expression of the paternally derived *UCL1_4*.*1k*::*GUS* transgene in a wild-type seed at 1 DAP. (K) Expression of the paternally derived *UCL1_2*.*7k*::*GUS* transgene in a wild-type seed at 1 DAP. (L) Expression of the paternally derived *UCL1_1*.*5k*::*GUS* transgene in a wild-type seed at 1 DAP. (M) Expression of the paternally derived *UCL1_5*.*2k*::*UCL1*:*GUS* transgene in a wild-type seed at 1 DAP. (N) Expression of the paternally derived *UCL1_4*.*1k*::*UCL1*:*GUS* transgene in a wild-type seed at 1 DAP. (O) Expression of the paternally derived *UCL1_2*.*7k*::*UCL1*:*GUS* transgene in a wild-type seed at 1 DAP. (P) Expression of the paternally derived *UCL1_1*.*5k*::*UCL1*:*GUS* transgene in a wild-type seed at 1 DAP. Scale bars: 50 μm.
(PDF)

S4 Fig Bi-allelic expression of the *GUS* transgene driven by various *UCL1* promoter regions shorter than 2.0 kb. (A-D) Ovules expressing the *GUS* transgene driven by various *UCL1* promoter fragments after emasculation. (A) Expression of the maternally derived *UCL1_2*.*0k*::*GUS* transgene in an ovule. (B) Expression of a maternally derived *UCL1_1*.*9k*::*GUS* transgene in an ovule. (C) Expression of a maternally derived *UCL1_1*.*7k*::*GUS* transgene in an ovule. (D) Expression of a maternally derived *UCL1_1*.*0k*::*GUS* transgene in an ovule. (E-H) Seeds expressing the *GUS* transgene driven by various fragments of the *UCL1* promoter after self-pollination. (E) Expression of the *UCL1_2*.*0k*::*GUS* transgene in a wild-type seed at 1 DAP. (F) Expression of the *UCL1_1*.*9k*::*GUS* transgene in a wild-type seed at 1 DAP. (G) Expression of the *UCL1_1*.*7k*::*GUS* transgene in a wild-type seed at 1 DAP. (H) Expression of the *UCL1_1*.*0k*::*GUS* transgene in a wild-type seed at 1 DAP. (I-L) Seeds of *UCL1*::*GUS* transgenic plants pollinated with wild-type pollen. (I) Expression of the maternally derived *UCL1_2*.*0k*::*GUS* transgene in a wild-type seed at 1 DAP. (J) Expression of the maternally derived *UCL1_1*.*9k*::*GUS* transgene in a wild-type seed at 1 DAP. (K) Expression of the maternally derived *UCL1_1*.*7k*::*GUS* transgene in a wild-type seed at 1 DAP. (L) Expression of the maternally derived *UCL1_1*.*0k*::*GUS* transgene in a wild-type seed at1 DAP.
(PDF)

S5 Fig Inheritance of paternal *dme-2* or *fie-1* does not affect the *UCL1* imprinting pattern. (A) Analysis of allele-specific expression of *UCL1* using the CAPS primers in S2 Table. RT-PCR was performed using endosperm RNA isolated from the products of crosses between RLD stigmas and *dme-2* or *fie-1* pollen at 3 DAP or 4 DAP. The RT-PCR products were analyzed before and after *Eco*RI digestion. (B) Sequencing chromatograms at the SNP regions showing allele-specific expression. The RT-PCR products amplified from endosperm RNA isolated from the products of crosses between L*er* stigmas and RLD pollen, *fie-1* stigmas and RLD pollen, and RLD stigmas and *fie-1* pollen were sequenced.
(PDF)

S6 Fig Mutations in genes involved in non-CG methylation do not affect the maternal silencing of *UCL1*. (A-D) Ovules after emasculation. (A) Expression of the maternally derived *UCL1_4*.*1k*::*GUS* transgene in a wild-type ovule. (B) Expression of the maternally derived *UCL1_4*.*1k*::*GUS* transgene in an *ago4–1* mutant ovule. (C) Expression of the maternally derived *UCL1_4*.*1k*::*GUS* transgene in a *rdr2–1* mutant ovule. (D) Expression of the maternally derived *UCL1_4*.*1k*::*GUS* transgene in a *dcl3–1* mutant ovule. (E-L) Seeds from plants hemizygous for the *GUS* transgene and heterozygous for *ago4–1*, *rdr2–1*, and *dcl3–1*. The *ago4–1*, *rdr2–1*, and *dcl3–1* mutants were used as females in crosses with wild-type pollen. (E) Expression of the maternally derived *UCL1_4*.*1k*::*GUS* transgene in a wild-type seed at 1 DAP. (F) Expression of the maternally derived *UCL1_4*.*1k*::*GUS* transgene in a *ago4–1* mutant seed at 1 DAP. (G) Expression of the maternally derived *UCL1_4*.*1k*::*GUS* transgene in a *rdr2–1* mutant seed at 1 DAP. (H) Expression of the maternally derived *UCL1_4*.*1k*::*GUS* transgene in a *dcl3–1* mutant seed at 1 DAP. (I) Expression of the maternally derived *UCL1_4*.*1k*::*UCL1*:*GUS* transgene in a wild-type seed at 1 DAP. (J) Expression of the maternally derived *UCL1_4*.*1k*::*UCL1*:*GUS* transgene in a *ago4–1* mutant seed at 1 DAP. (K) Expression of the maternally derived *UCL1_4*.*1k*::*UCL1*:*GUS* transgene in a *rdr2–1* mutant seed at 1 DAP. (L) Expression of the maternally derived *UCL1_4*.*1k*::*UCL1*:*GUS* transgene in a *dcl3–1* mutant seed at 1 DAP. Scale bars: 50 μm.
(PDF)

S1 Table Publicly available CpG methylation pattern of the *UCL1* 5′ upstream region in the wild-type endosperm, *dme-2* endosperm and wild-type embryo. (DOC)

S2 Table Sequences of primers used in this study. (DOC)
