# Supplementary figures and images for: Control of Paternally Expressed Imprinted UPWARD CURLY LEAF1, a Gene Encoding an F-Box Protein That Regulates CURLY LEAF Polycomb Protein, in the Arabidopsis Endosperm
Source: PLoS One. 2015 Feb 17;10(2):e0117431. doi: 10.1371/journal.pone.0117431 (PMC4331533; doi:10.1371/journal.pone.0117431)

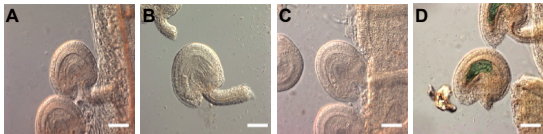

before  
fertilization

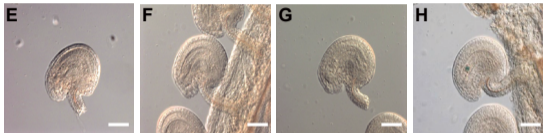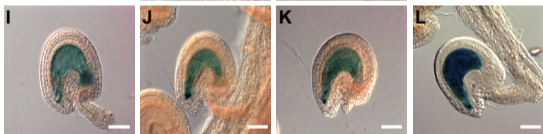

after  
selfing

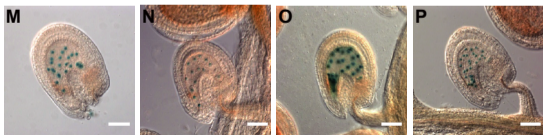

5.2 kb

4.1 kb

2.7 kb

1.5 kb

Supplement: S2 Fig — (A-H) Ovules expressing GUS transgenes driven by various lengths of the UCL1 promoter after emasculation. (A) Expression of the maternally derived UCL1_5.2k::GUS transgene in a wild-type ovule. (B) Expression of the maternally derived UCL1_4.1k::GUS transgene in a wild-type ovule. (C) Expression of the maternally derived UCL1_2.7k::GUS transgene in a wild-type ovule. (D) Expression of the maternally derived UCL1_1.5k::GUS transgene in a wild-type ovule. (E) Expression of the maternally derived UCL1_5.2k::UCL1:GUS transgene in a wild-type ovule. (F) Expression of the maternally derived UCL1_4.1k::UCL1:GUS transgene in a wild-type ovule. (G) Expression of the maternally derived UCL1_2.7k::UCL1:GUS transgene in a wild-type ovule. (H) Expression of the maternally derived UCL1_1.5k::UCL1:GUS transgene in a wild-type ovule. (I-P) Seeds expressing GUS transgenes driven by various lengths of the UCL1 promoter after self- pollination. (I) Expression of the UCL1_5.2k::GUS transgene in a wild-type seed at 1 DAP. (J) Expression of the UCL1_4.1k::GUS transgene in a wild-type seed at 1 DAP. (K) Expression of the UCL1_2.7k::GUS transgene in a wild-type seed at 1 DAP. (L) Expression of the UCL1_1.5k::GUS transgene in a wild-type seed at 1 DAP. (M) Expression of the UCL1_5.2k::UCL1:GUS transgene in a wild-type seed at 1 DAP. (N) Expression of the UCL1_4.1k::UCL1:GUS transgene in a wild-type seed at 1 DAP. (O) Expression of the UCL1_2.7k::UCL1:GUS transgene in a wild-type seed at 1 DAP. (P) Expression of the UCL1_1.5k::UCL1:GUS transgene in a wild-type seed at 1 DAP. Scale bars: 50 μm. (PDF) [file pone.0117431.s002.pdf]

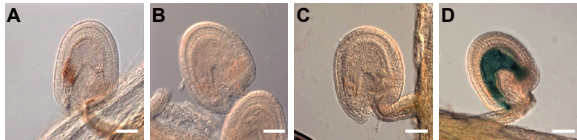

*UCL1*<sup>♀</sup>

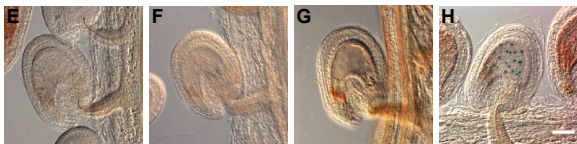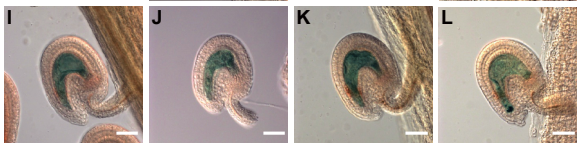

*UCL1*<sup>♂</sup>

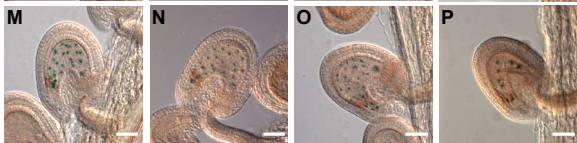

5.2 kb

4.1 kb

2.7 kb

1.5 kb

Supplement: S3 Fig — (A-D, I-L) Seeds of reciprocal crosses between the UCL1::GUS transgenic plant and Col-0 wild type. (E-H, M-P) Seeds of reciprocal crosses between the UCL1_4.1k::UCL1:GUS transgenic plant and the Col-0 wild type. (A) Expression of the maternally derived UCL1_5.2k::GUS transgene in a wild-type seed at 1 DAP. (B) Expression of the maternally derived UCL1_4.1k::GUS transgene in a wild-type seed at 1 DAP. (C) Expression of the maternally derived UCL1_2.7k::GUS transgene in a wild-type seed at 1 DAP. (D) Expression of the maternally derived UCL1_1.5k::GUS transgene in a wild-type seed at 1 DAP. (E) Expression of the maternally derived UCL1_5.2k::UCL1:GUS transgene in a wild-type seed at 1 DAP. (F) Expression of the maternally derived UCL1_4.1k::UCL1:GUS transgene in wild-type seed at 1 DAP. (G) Expression of the maternally derived UCL1_2.7k::UCL1:GUS transgene in a wild-type seed at 1 DAP. (H) Expression of the maternally derived UCL1_1.5k::UCL1:GUS transgene in a wild-type seed at 1 DAP. (I) Expression of the paternally derived UCL1_5.2k::GUS transgene in a wild-type seed at 1 DAP. (J) Expression of the paternally derived UCL1_4.1k::GUS transgene in a wild-type seed at 1 DAP. (K) Expression of the paternally derived UCL1_2.7k::GUS transgene in a wild-type seed at 1 DAP. (L) Expression of the paternally derived UCL1_1.5k::GUS transgene in a wild-type seed at 1 DAP. (M) Expression of the paternally derived UCL1_5.2k::UCL1:GUS transgene in a wild-type seed at 1 DAP. (N) Expression of the paternally derived UCL1_4.1k::UCL1:GUS transgene in a wild-type seed at 1 DAP. (O) Expression of the paternally derived UCL1_2.7k::UCL1:GUS transgene in a wild-type seed at 1 DAP. (P) Expression of the paternally derived UCL1_1.5k::UCL1:GUS transgene in a wild-type seed at 1 DAP. Scale bars: 50 μm. (PDF) [file pone.0117431.s003.pdf]

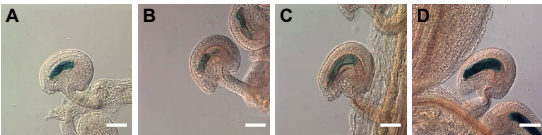

before  
fertilization

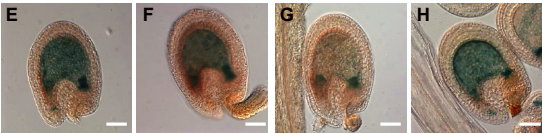

*selfing*

after  
fertilization

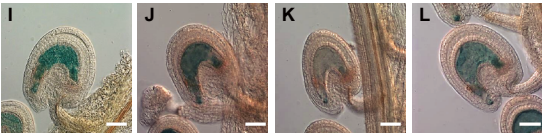

*UCL1*<sup>♀</sup>

2.0 kb

1.9 kb

1.7 kb

1.0 kb

Supplement: S4 Fig — (A-D) Ovules expressing the GUS transgene driven by various UCL1 promoter fragments after emasculation. (A) Expression of the maternally derived UCL1_2.0k::GUS transgene in an ovule. (B) Expression of a maternally derived UCL1_1.9k::GUS transgene in an ovule. (C) Expression of a maternally derived UCL1_1.7k::GUS transgene in an ovule. (D) Expression of a maternally derived UCL1_1.0k::GUS transgene in an ovule. (E-H) Seeds expressing the GUS transgene driven by various fragments of the UCL1 promoter after self-pollination. (E) Expression of the UCL1_2.0k::GUS transgene in a wild-type seed at 1 DAP. (F) Expression of the UCL1_1.9k::GUS transgene in a wild-type seed at 1 DAP. (G) Expression of the UCL1_1.7k::GUS transgene in a wild-type seed at 1 DAP. (H) Expression of the UCL1_1.0k::GUS transgene in a wild-type seed at 1 DAP. (I-L) Seeds of UCL1::GUS transgenic plants pollinated with wild-type pollen. (I) Expression of the maternally derived UCL1_2.0k::GUS transgene in a wild-type seed at 1 DAP. (J) Expression of the maternally derived UCL1_1.9k::GUS transgene in a wild-type seed at 1 DAP. (K) Expression of the maternally derived UCL1_1.7k::GUS transgene in a wild-type seed at 1 DAP. (L) Expression of the maternally derived UCL1_1.0k::GUS transgene in a wild-type seed at1 DAP. (PDF) [file pone.0117431.s004.pdf]

**A**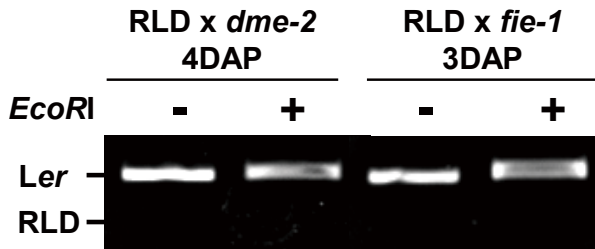**B**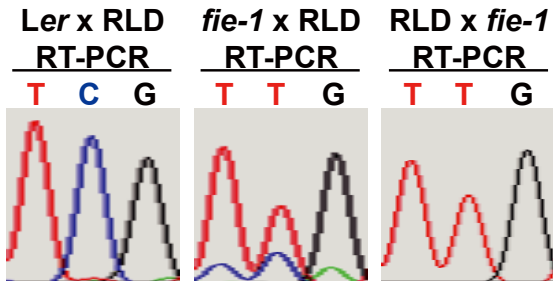

Supplement: S5 Fig — (A) Analysis of allele-specific expression of UCL1 using the CAPS primers in S2 Table. RT-PCR was performed using endosperm RNA isolated from the products of crosses between RLD stigmas and dme-2 or fie-1 pollen at 3 DAP or 4 DAP. The RT-PCR products were analyzed before and after EcoRI digestion. (B) Sequencing chromatograms at the SNP regions showing allele-specific expression. The RT-PCR products amplified from endosperm RNA isolated from the products of crosses between Ler stigmas and RLD pollen, fie-1 stigmas and RLD pollen, and RLD stigmas and fie-1 pollen were sequenced. (PDF) [file pone.0117431.s005.pdf]

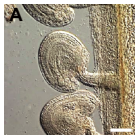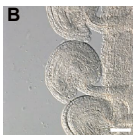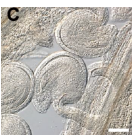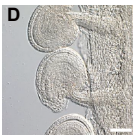

before  
fertilization

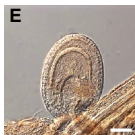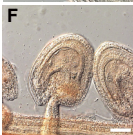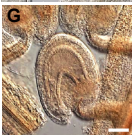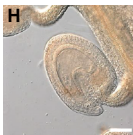

*UCL1*<sup>♀</sup>

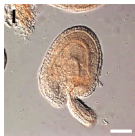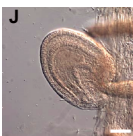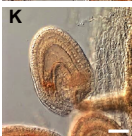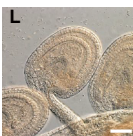

WT

*ago4-1*

*rdr2-1*

*dcl3-1*

Supplement: S6 Fig — (A-D) Ovules after emasculation. (A) Expression of the maternally derived UCL1_4.1k::GUS transgene in a wild-type ovule. (B) Expression of the maternally derived UCL1_4.1k::GUS transgene in an ago4–1 mutant ovule. (C) Expression of the maternally derived UCL1_4.1k::GUS transgene in a rdr2–1 mutant ovule. (D) Expression of the maternally derived UCL1_4.1k::GUS transgene in a dcl3–1 mutant ovule. (E-L) Seeds from plants hemizygous for the GUS transgene and heterozygous for ago4–1, rdr2–1, and dcl3–1. The ago4–1, rdr2–1, and dcl3–1 mutants were used as females in crosses with wild-type pollen. (E) Expression of the maternally derived UCL1_4.1k::GUS transgene in a wild-type seed at 1 DAP. (F) Expression of the maternally derived UCL1_4.1k::GUS transgene in a ago4–1 mutant seed at 1 DAP. (G) Expression of the maternally derived UCL1_4.1k::GUS transgene in a rdr2–1 mutant seed at 1 DAP. (H) Expression of the maternally derived UCL1_4.1k::GUS transgene in a dcl3–1 mutant seed at 1 DAP. (I) Expression of the maternally derived UCL1_4.1k::UCL1:GUS transgene in a wild-type seed at 1 DAP. (J) Expression of the maternally derived UCL1_4.1k::UCL1:GUS transgene in a ago4–1 mutant seed at 1 DAP. (K) Expression of the maternally derived UCL1_4.1k::UCL1:GUS transgene in a rdr2–1 mutant seed at 1 DAP. (L) Expression of the maternally derived UCL1_4.1k::UCL1:GUS transgene in a dcl3–1 mutant seed at 1 DAP. Scale bars: 50 μm. (PDF) [file pone.0117431.s006.pdf]
