# Supplementary material for: Control of Paternally Expressed Imprinted UPWARD CURLY LEAF1, a Gene Encoding an F-Box Protein That Regulates CURLY LEAF Polycomb Protein, in the Arabidopsis Endosperm
Source: PLoS One. 2015 Feb 17;10(2):e0117431. doi: 10.1371/journal.pone.0117431 (PMC4331533; doi:10.1371/journal.pone.0117431)
Supplement: S1 Table — (DOC) [file pone.0117431.s007.doc]

Table S1. Publicly available CG methylation pattern of the *UCL1* 5’ upstream region in the wild-type endosperm, *dme-2* endosperm, and wild-type embryo.

| **Position** | **endo_wt_CG** | **endo_dme_CG** | **emb_wt_CG** | **p_wt_dme-CG** | **p_endo_emb-CG** |
| --- | --- | --- | --- | --- | --- |
| -2269 | 0.129 | 0.5938 | 0.4545 | 0.000116 | 0.0326017 |
| -2151 | 0.1 | 0.8571 | 1 | 0.000365 | 0.0454545 |
| -2047 | 0.3333 | 0.7692 | 1 | 0.016282 | 0.00309598 |
| -2012 | 0.2353 | 0.6176 | 0.8571 | 0.008907 | 0.00072064 |
| -1905 | 0.1481 | 0.5714 | 0.6 | 3.17E-05 | 0.00087306 |
| -1836 | 0.6528 | 0.9167 | 0.8966 | 0.007916 | 0.00806465 |
| -1819 | 0.5833 | 0.8519 | 1 | 0.007524 | 1.65E-05 |
| -1774 | 0.2581 | 0.4211 | 0.7391 | 0.042508 | 6.86E-05 |
| -1771 | 0.0345 | 0.64 | 0.6471 | 1.26E-06 | 9.22E-06 |
| -1693 | 0.1132 | 0.561 | 0.85 | 5.25E-08 | 9.00E-11 |
| -1627 | 0.0446 | 0.2326 | 0.2857 | 0.000945 | 0.00192939 |
| -1598 | 0.5645 | 0.8298 | 0.9062 | 0.000627 | 0.00013677 |
| -1586 | 0.6783 | 0.9565 | 0.875 | 5.15E-05 | 0.0149823 |
| -1575 | 0.7615 | 0.8913 | 1 | 0.032166 | 0.00196389 |
| -1491 | 0.4286 | 0.0455 | 0.4 | 0.000892 | 0.362303 |
| -839 | 0.3125 | 0.8649 | 1 | 0.000128 | 0.0017499 |
| -694 | 0.2308 | 0.8788 | 1 | 4.36E-07 | 7.26E-08 |
| -686 | 0.2 | 0.6364 | 1 | 0.02394 | 0.034965 |
| -685 | 0.0435 | 0.6944 | 0.875 | 3.49E-07 | 2.33E-05 |
