# Supplementary material for: Control of Paternally Expressed Imprinted UPWARD CURLY LEAF1, a Gene Encoding an F-Box Protein That Regulates CURLY LEAF Polycomb Protein, in the Arabidopsis Endosperm
Source: PLoS One. 2015 Feb 17;10(2):e0117431. doi: 10.1371/journal.pone.0117431 (PMC4331533; doi:10.1371/journal.pone.0117431)
Supplement: S2 Table — (DOC) [file pone.0117431.s008.doc]

Table S2. Sequences of primers used in this study

| **Purpose** | **Label** | **Oligo name** | **Sequence** |
| --- | --- | --- | --- |
| **qRT-PCR** | JCW118 | q-65740-L | CTC TCA CGTGCA GCC TTC TT |
|  | JCW119 | q-65740-R | TGA TTA TCC AGC CCT TAC TCG |
|  | JCW480 | q-65740_1 F | TTG TGA TGG CTA CCG ATA CTT G |
|  | JCW481 | q-658740_1 R | CGA CTT TCT GGT GAT ACT ACT TCC |
|  | JCW564 | q-UCL1-2-S | GCA GCC TTC TTC CGA GTC |
|  | JCW565 | q-UCL1-2-AS | AGA GAG GGT TGA GGA GAT GG |
|  | JCW566 | q-UCL1-3-S | CGG CGT GAA GAA GAA TCC |
|  | JCW567 | q-UCL1-3-AS | AGT GAC TCG GAA GAA GGC |
|  | JCW633 | q-TUB2-L | ATC GAT TCC GTT CTC GAT GT |
|  | JCW634 | q-TUB2-R | ATC CAG TTC CTC CTC CCA AC |
|  | JCW635 | q-ACT7-L | CGC TGC TTC TCG AAT CTT CT |
|  | JCW636 | q-ACT7-R | CCA TTC CAG TTC CAT TGT CA |
| **Cloning** | JCW615 | 1._Sal_40PRO_5.2K_F | ACG CGT CGA CGG TAA GTG ACC GGG AGA CAA |
|  | JCW616 | 2._Sal_40PRO_4.1K_F | ACG CGT CGA CAA GCA GAC CGC ACT GAG AAT |
|  | JCW617 | 3._Sal_40PRO_2.7K_F | ACG CGT CGA CCA ACC CTT ACT CCC TTT CTT TC |
|  | JCW618 | 4._Sal_40PRO_1.5K_F | ACG CGT CGA CCC ATC CCT CAC TTG GTT TCC CA |
|  | JCW619 | 5._Bam_40Pro_R | CGC GGA TCC TTT GCT ACT TTG ATT GTT TGT GAT |
|  | JCW620 | 6._Bam_40PnC_R | CGC GGA TCC AAG AAA GCT AGG AAA AAA CAT TTC |
|  | JCW652 | 11_Sal_40PRO_20K_F | ACG CGT CGA CTC TCC TAC ATT TTA TTT AAT AAC GA |
|  | JCW653 | 12_Sal_40PRO_19K_F | ACG CGT CGA CAA CAT ACA TTA AAT AAC TGA AAA CC |
|  | JCW654 | 13_Sal_40PRO_17K_F | ACG CGT CGA CAA TCC ATC TCC AAC ACC ACC CT |
|  | JCW655 | 14_Sal_40PRO_10K_F | ACG CGT CGA CTG ATT GAT TTT ATG AGT TTT CAC AT |
|  | JCW656 | 15_Sal_40PRO_09K_F | ACG CGT CGA CGT TTA TGA TTC GTC ATA ATT AAT TA |
| **Genotyping** | JCW637 | 1_At1g65760_genoF | GAC AAA GCT AGG ATA AAA CAT TTC G |
|  | JCW638 | 2_At1g65760_genoR | GGT TGA TTG CGA TTG GTC TAA |
|  | JCW639 | 3_At1g65750_genoF | TCG AGG AGG AGG AGA GAG TG |
|  | JCW640 | 4_At1g65750_genoR | TCA ATC ATT CCA AAA CAG AGA GA |
| **(d)CAPS** | JCW641 | 5_UCL1_CAPs_Eco_F | TCG GAA TCG GTA GGG ATG |
| **marker** | JCW642 | 6_UCL1_CAPs_Eco_R | CTT TGG GGA GGC GTT TGA |
|  | JCW643 | 7_met1-dCAPS_  1424dBgI | TGT GAC TGA GAA CCG CTG TCA GGA TCG TTT AAG GAG ATC |
|  | JCW644 | 8_met1-dCAPS_1424F | CGT ACT ATA AGA CCT CCG AAG |
