# Supplementary material for: Control of Paternally Expressed Imprinted UPWARD CURLY LEAF1, a Gene Encoding an F-Box Protein That Regulates CURLY LEAF Polycomb Protein, in the Arabidopsis Endosperm
Source: PLoS One. 2015 Feb 17;10(2):e0117431. doi: 10.1371/journal.pone.0117431 (PMC4331533; doi:10.1371/journal.pone.0117431)
Supplement: S1 Fig — Identical nucleotides in different ecotypes are indicated with asterisks and distinguishable SNPs are indicated with dots and red boxes. The Col-0, Ler, and En-2 ecotypes have identical UCL sequences, while the RLD and C24 ecotypes have polymorphic sites. (PDF) [file pone.0117431.s001.pdf]

```
Col_qRT900-q65470_L.txt          CTAGCCCTTCTTCTCTCTCGAGTAGGGCTGGATAATCAAATCCGACATGG
Ler_qRT900-q65470_L.txt          --TAGCCTTTTTCTCTCTCGAGTAGGGCTGGATAATCAAATCCGACATGG
RLD_qRT_900-q65470_L.txt         ---CTAGGATCTCTCTCTCGAGTAGGGCTGGATAATCAAATCCGACTTGG
C24_qRT900-q65470_L.txt          ACTACGACTTCTCTCTCTCGAGTAGGGCTGGATAATCAAATCCGACTTGG
En2_qRT900-q65470_L.txt          ---NTAGACTCTCTCTCTCGAGTAGGGCTGGATAATCAAATCCGACATGG
                                            ***********************************:***

Col_qRT900-q65470_L.txt          ACACCAACTCCGGGAGATTCCATCTCCTCAACCCTCTCTCACGTTTTCCA
Ler_qRT900-q65470_L.txt          ACACCAACTCCGGGAGATTCCATCTCCTCAACCCTCTCTCACGTTTTCCA
RLD_qRT_900-q65470_L.txt         ACACCAACTCCGGGAGATTCCGTCTCCTCAACCCTCTCTCACGTTTTCCA
C24_qRT900-q65470_L.txt          ACACCAACTCCGGGAGATTCCGTCTCCTCAACCCTCTCTCACGTTTTCCA
En2_qRT900-q65470_L.txt          ACACCAACTCCGGGAGATTCCATCTCCTCAACCCTCTCTCACGTTTTCCA
                                 *********************.****************************

Col_qRT900-q65470_L.txt          TTGCGTATTTCTTCCGAGAGCCTTGATCTTTTGGATTTCACTGTCTCCGA
Ler_qRT900-q65470_L.txt          TTGCGTATTTCTTCCGAGAGCCTTGATCTTTTGGATTTCACTGTCTCCGA
RLD_qRT_900-q65470_L.txt         TTGCGTATTTCTTCCGAGAGCCTTGATCTTTTGGATTTCACTGTCTCCGA
C24_qRT900-q65470_L.txt          TTGCGTATTTCTTCCGAGAGCCTTGATCTTTTGGATTTCACTGTCTCCGA
En2_qRT900-q65470_L.txt          TTGCGTATTTCTTCCGAGAGCCTTGATCTTTTGGATTTCACTGTCTCCGA
                                 **************************************************

Col_qRT900-q65470_L.txt          GATTCAAGAATCTTATGCGGTTCTTAAGGACGCCAAGGGAAGACTACCGA
Ler_qRT900-q65470_L.txt          GATTCAAGAATCTTATGCGGTTCTTAAGGACGCCAAGGGAAGACTACCGA
RLD_qRT_900-q65470_L.txt         GATTCAAGAATCTTATGCGGTTCTTAAGGACGCCAAGGGAAGACTACCGA
C24_qRT900-q65470_L.txt          GATTCAAGAATCTTATGCGGTTCTTAAGGACGCCAAGGGAAGACTACCGA
En2_qRT900-q65470_L.txt          GATTCAAGAATCTTATGCGGTTCTTAAGGACGCCAAGGGAAGACTACCGA
                                 **************************************************

Col_qRT900-q65470_L.txt          ATCCAGGATACCAAAGAAGTGCTCTTGTCAAAGTGAAGGAAGGAGATGAT
Ler_qRT900-q65470_L.txt          ATCCAGGATACCAAAGAAGTGCTCTTGTCAAAGTGAAGGAAGGAGATGAT
RLD_qRT_900-q65470_L.txt         ATCCAGGATACCAAAGAAGTGCTCTTGTCAAAGTGAAGGAAGGAGAGGAT
C24_qRT900-q65470_L.txt          ATCCAGGATACCAAAGAAGTGCTCTTGTCAAAGTGAAGGAAGGAGAGGAT
En2_qRT900-q65470_L.txt          ATCCAGGATACCAAAGAAGTGCTCTTGTCAAAGTGAAGGAAGGAGATGAT
                                 ********************************************** ***

Col_qRT900-q65470_L.txt          CATCATCATGGAATTCTCGGAATCGGTAGGGATGGAACAATCAACTACTG
Ler_qRT900-q65470_L.txt          CATCATCATGGAATTCTCGGAATCGGTAGGGATGGAACAATCAACTACTG
RLD_qRT_900-q65470_L.txt         CATCATCATGGAATTCTCGGAATCGGTAGGGATGGAACAATCAACTACTG
C24_qRT900-q65470_L.txt          CATCATCATGGAATTCTCGGAATCGGTAGGGATGGAACAATCAACTACTG
En2_qRT900-q65470_L.txt          CATCATCATGGAATTCTCGGAATCGGTAGGGATGGAACAATCAACTACTG
                                 **************************************************

Col_qRT900-q65470_L.txt          GAATGGAAATGTCTTGAACGGATTCAAACAAATGGGTCATCACTTCTCCG
Ler_qRT900-q65470_L.txt          GAATGGAAATGTCTTGAACGGATTCAAACAAATGGGTCATCACTTCTCCG
RLD_qRT_900-q65470_L.txt         GAATGGAAATGTCTTGAACGAATTCAAACGAATGGGTCATCACTTCTCCG
C24_qRT900-q65470_L.txt          GAATGGAAATGTCTTGAACGAATTCAAACGAATGGGTCATCACTTCTCCG
En2_qRT900-q65470_L.txt          GAATGGAAATGTCTTGAACGGATTCAAACAAATGGGTCATCACTTCTCCG
                                 ********************.*********.*******************

Col_qRT900-q65470_L.txt          ACATTATAGTTCACAAAGGAGTAACTTACGTCTTGGATTCAAAAGGCATC
Ler_qRT900-q65470_L.txt          ACATTATAGTTCACAAAGGAGTAACTTACGTCTTGGATTCAAAAGGCATC
RLD_qRT_900-q65470_L.txt         ACATTATAGTTCACAAAGGAGTAACTTACGTCTTGGATTCAAAAGGCATC
C24_qRT900-q65470_L.txt          ACATTATAGTTCACAAAGGAGTAACTTACGTCTTGGATTCAAAAGGCATC
En2_qRT900-q65470_L.txt          ACATTATAGTTCACAAAGGAGTAACTTACGTCTTGGATTCAAAAGGCATC
                                 **************************************************

Col_qRT900-q65470_L.txt          GTTTGGTGCATTAATTCCGATCTTGAAATGTCAAGGTACGAAACTTCACT
Ler_qRT900-q65470_L.txt          GTTTGGTGCATTAATTCCGATCTTGAAATGTCAAGGTACGAAACTTCACT
RLD_qRT_900-q65470_L.txt         GTTTGGTGCATTAATTCCGATCTTGAAATGTCAAGGTACGAAACTTCACT
C24_qRT900-q65470_L.txt          GTTTGGTGCATTAATTCCGATCTTGAAATGTCAAGGTACGAAACTTCACT
En2_qRT900-q65470_L.txt          GTTTGGTGCATTAATTCCGATCTTGAAATGTCAAGGTACGAAACTTCACT
                                 **************************************************

Col_qRT900-q65470_L.txt          CGATGAAAACATGACTAATGGCTGTAGGAGATATTACATGAGATTTGTGG
Ler_qRT900-q65470_L.txt          CGATGAAAACATGACTAATGGCTGTAGGAGATATTACATGAGATTTGTGG
RLD_qRT_900-q65470_L.txt         CGATGAAAACATGACTAATGGCTGTAGGAGATATTACATGAGATTTGTGG
C24_qRT900-q65470_L.txt          CGATGAAAACATGACTAATGGCTGTAGGAGATATTACATGAGATTTGTGG
En2_qRT900-q65470_L.txt          CGATGAAAACATGACTAATGGCTGTAGGAGATATTACATGAGATTTGTGG
                                 **************************************************

Col_qRT900-q65470_L.txt          ATTGCTGTGGAGAGCTTTACGTTATCAAACGCCTCCCCAAAGAGAATTCT
Ler_qRT900-q65470_L.txt          ATTGCTGTGGAGAGCTTTACGTTATCAAACGCCTCCCCAAAGAGAATTCT
RLD_qRT_900-q65470_L.txt         ATTGCTGTGGAGAGCTTTACGTTATCAAACGCCTCCCCAAAGAGAATTCT
C24_qRT900-q65470_L.txt          ATTGCTGTGGAGAGCTTTACGTTATCAAACGCCTCCCCAAAGAGAATTCT
En2_qRT900-q65470_L.txt          ATTGCTGTGGAGAGCTTTACGTTATCAAACGCCTCCCCAAAGAGAATTCT
                                 **************************************************

Col_qRT900-q65470_L.txt          CGAAAGAGAAAATCGACCTTATTTCAATTTTCAAGAACGGCTGGTTTCAA
Ler_qRT900-q65470_L.txt          CGAAAGAGAAAATCGACCTTATTTCAATTTTCAAGAACGGCTGGTTTCAA
RLD_qRT_900-q65470_L.txt         CGAAAGAGAAAATCGACCTTATTTCAATTTTCAAGAACGGCTGGTTTCAA
C24_qRT900-q65470_L.txt          CGAAAGAGAAAATCGACCTTATTTCAATTTTCAAGAACGGCTGGTTTCAA
En2_qRT900-q65470_L.txt          CGAAAGAGAAAATCGACCTTATTTCAATTTTCAAGAACGGCTGGTTTCAA
                                 **************************************************

Col_qRT900-q65470_L.txt          GGTTTACAAGATAGATAAGGAATTAGCCAAATGGGTAGAAGTTAAGACCT
Ler_qRT900-q65470_L.txt          GGTTTACAAGATAGATAAGGAATTAGCCAAATGGGTAGAAGTTAAGACCT
RLD_qRT_900-q65470_L.txt         GGTTTACAAGATAGATAAGGAATTAGCAAAATGGATAGAAGTTAAGACCT
C24_qRT900-q65470_L.txt          GGTTTACAAGATAGATAAGGAATTAGCAAAATGGATAGAAGTTAAGACCT
En2_qRT900-q65470_L.txt          GGTTTACAAGATAGATAAGGAATTAGCCAAATGGGTAGAAGTTAAGACCT
                                 ***************************.******.***************

Col_qRT900-q65470_L.txt          TGGGAGACAATGCATTTGTGATGGCTACCGATACTTGTTTCTCGGTTTTG
Ler_qRT900-q65470_L.txt          TGGGAGACAATGCATTTGTGATGGCTACCGATACTTGTTTCTCGGTTTTG
RLD_qRT_900-q65470_L.txt         TGGGAGACAATGCATTTGTGATGGCTACCGATACTTGTTTCTCGGCTTTG
C24_qRT900-q65470_L.txt          TGGGAGACAATGCATTTGTGATGGCTACCGATACTTGTTTCTCGGCTTTG
En2_qRT900-q65470_L.txt          TGGGAGACAATGCATTTGTGATGGCTACCGATACTTGTTTCTCGGTTTTG
                                 ********************************************* ****
```
